# Quantifying uncertainty about future antimicrobial resistance: Comparing structured expert judgment and statistical forecasting methods

**DOI:** 10.1371/journal.pone.0219190

**Published:** 2019-07-05

**Authors:** Abigail R. Colson, Itamar Megiddo, Gerardo Alvarez-Uria, Sumanth Gandra, Tim Bedford, Alec Morton, Roger M. Cooke, Ramanan Laxminarayan

**Affiliations:** 1 Department of Management Science, University of Strathclyde, Glasgow, Scotland, United Kingdom; 2 Center for Disease Dynamics, Economics & Policy, Washington, DC, United States of America; 3 Department of Infectious Diseases, Rural Development Trust Hospital, Bathalapalli, Andhra Pradesh, India; 4 Resources for the Future, Washington, DC, United States of America; 5 TU Delft, Delft, The Netherlands; 6 Princeton Environmental Institute, Princeton University, Princeton, New Jersey, United States of America; University of Sheffield, UNITED KINGDOM

## Abstract

The increase of multidrug resistance and resistance to last-line antibiotics is a major global public health threat. Although surveillance programs provide useful current and historical information on the scale of the problem, the future emergence and spread of antibiotic resistance is uncertain, and quantifying this uncertainty is crucial for guiding decisions about investment in antibiotics and resistance control strategies. Mathematical and statistical models capable of projecting future rates are challenged by the paucity of data and the complexity of the emergence and spread of resistance, but experts have relevant knowledge. We use the Classical Model of structured expert judgment to elicit projections with uncertainty bounds of resistance rates through 2026 for nine pathogen-antibiotic pairs in four European countries and empirically validate the assessments against data on a set of calibration questions. The performance-weighted combination of experts in France, Spain, and the United Kingdom projected that resistance for five pairs on the World Health Organization’s priority pathogens list (*E*. *coli* and *K*. *pneumoniae* resistant to third-generation cephalosporins and carbapenems and MRSA) would remain below 50% in 2026. In Italy, although upper bounds of 90% credible ranges exceed 50% resistance for some pairs, the medians suggest Italy will sustain or improve its current rates. We compare these expert projections to statistical forecasts based on historical data from the European Antimicrobial Resistance Surveillance Network (EARS-Net). Results from the statistical models differ from each other and from the judgmental forecasts in many cases. The judgmental forecasts include information from the experts about the impact of current and future shifts in infection control, antibiotic usage, and other factors that cannot be easily captured in statistical forecasts, demonstrating the potential of structured expert judgment as a tool for better understanding the uncertainty about future antibiotic resistance.

## Introduction

The U.S. Centers for Disease Control and Prevention (CDC) estimates that antibiotic-resistant pathogens cause more than 2 million infections and 23,000 deaths in the United States annually [1], and its European counterpart estimates that 25,000 deaths in Europe every year are due to antibiotic resistance [2]. Antibiotic resistance is increasing around the world, in both high- and low-income countries, and to first-line and last-resort antibiotics [3]. In response to growing antibiotic resistance, world leaders have discussed the issue at meetings of the G7, G20, and United Nations General Assembly [4–7], and public investment in research targeting the problem exceeds $1 billion annually [8,9]. Understanding the future risk of antibiotic resistance is important to guide high-level policy and investments addressing the problem.

Antibiotic resistance surveillance programs provide crucial information on the current state of the problem, underscore the need for action, and identify current priority targets [10,11], but surveillance programs can only describe the past and current situation. Due to the long timeframe required to identify, develop, and bring new antibiotics to market, decisions about prioritizing and investing in antibiotic development must consider the future trajectory of resistance in addition to the current situation. However, the future emergence and spread of antibiotic resistance is uncertain, so quantifying this uncertainty is crucial for guiding decisions about investment in new antibiotics and resistance control strategies. Unfortunately, research on projected resistance rates is sparse. Researchers from different fields have used a variety of methodologies to investigate related questions, such as ecology and evolutionary biology models that predict and explain the emergence and spread of new resistance genes [12–16]; historical time series or before-and-after studies that look at past trends, correlations, or the impact of a specific policy or intervention [17–21]; and compartmental or simulation models that try to understand specific dynamics related to the spread of resistance or consider hypothetical scenarios [22–25]. Although essential for better understanding antibiotic resistance, none of that work aims to project or quantify uncertainty about future rates resistance. Some studies use time series methods to make short-term projections (e.g., six months) of resistance at a single healthcare facility or unit based on its history of antibiotic resistance and consumption (e.g. [26–31]). While useful for decision-making at a single facility, the results are not informative for policy at the national or global level.

One study used resistance and consumption data from 2001 to 2007 to project country-level annual rates of *Escherichia coli* resistance to fluoroquinolones through 2013 in 18 European countries and the United States [32], but the study assumed antibiotic consumption in each country was fixed, and there has been no follow-up on other pathogen-antibiotic combinations or updating the results for future years. The projections were based on data from the European Antimicrobial Resistance Surveillance System (EARSS), the predecessor to the current European Antimicrobial Surveillance Network (EARS-Net). The study’s projections typically overestimated resistance rates compared to what EARS-Net reported in 2013 [33]. The observed resistance rate in 2013 was below the projected rate in 17 of the 18 European countries studied and below the lower bound of the 95% confidence interval in 11 countries.

Mathematical models capable of projecting country-level resistance rates are challenged by the large number of interacting determinants of the emergence and spread of antibiotic resistance [34,35]. For example, an observed rise in resistance rates for a particular antibiotic may be followed by physicians prescribing it less, which influences the future resistance rate, implying resistance does not grow at a steady rate. Statistical forecasting methods struggle to process and cannot anticipate fundamental changes to the environment [36], so they may be problematic for projecting antibiotic resistance, given the shifting policy landscape around antibiotic use and infection control and the possibility of new drugs coming on the market or new resistance mechanisms emerging. Experts, however, have extensive domain knowledge about these issues that is relevant for thinking about the future trajectory of resistance and its uncertainty.

While all forecasting involves some expert judgment, for example, in the choice of data or model structure [37,38]. Experts can also directly supply forecasted values, a technique that is especially useful when historical data are not available or have limited predictive value [39]. Expert judgment can be subject to a range of biases [40], but a set of techniques called “expert elicitation” enable experts to provide estimates and quantify their uncertainty about parameters of interest—including projecting future events and values—in a way that minimizes opportunities for bias [38,41,42]. One such method, the Classical Model of structured expert judgment, has been used in over 80 applications, including estimating the burden of foodborne disease [43–45] and forecasting volcanic activity on the island of Montserrat [46–48], the introduction of invasive species in the Great Lakes [49], and future sea level rise caused by ice sheet melt [50]. Common across these application areas is the need to make evidence-based decisions but a lack of adequate data and/or models upon which to rely. The Classical Model is unique among expert elicitation techniques in that the performance of experts and their combinations is validated against empirical data [51], and the method itself has been extensively evaluated based on its performance predicting both in- and out-of-sample data [52–54]. This element of validation makes the method well-suited for expert judgment applications involving public policy.

In this study, we use the Classical Model to quantify uncertainty about the future rate of resistance for nine pathogen-antibiotic combinations (*Escherichia coli* and fluoroquinolones, *E*. *coli* and third-generation cephalosporins, *E*. *coli* and carbapenems, *Klebsiella pneumoniae* and third-generation cephalosporins, *K*. *pneumoniae* and carbapenems, *Staphylococcus aureus* and methicillin (MRSA), *Streptococcus pneumoniae* and penicillins, *Neisseria gonorrhoeae* and third-generation cephalosporins, and *Pseudomonas aeruginosa* and any available treatment) in four European countries (France, Italy, Spain, and the United Kingdom). We compare the results from the Classical Model to standard statistical forecasting approaches and discuss the strengths and weaknesses of both in the context of understanding the trajectory of and uncertainty about future rates of antibiotic resistance.

## Methods

### Expert elicitation and combination with the Classical Model

This study does not involve human participants. We conducted interviews with experts about their field of expertise. We did not use human subjects. Thus, ethics committee approval was not required for the study.

In the Classical Model, experts express their uncertainty about unknown quantities by providing specified quantiles from their subjective probability distributions [51]. Experts provide these assessments for two types of questions: variables of interest and calibration questions (also called “seed questions”). The variables of interest are the focus of the elicitation; they are questions that cannot be adequately addressed with existing data or models, so expert judgment is needed. Calibration questions are items which are closely related to the variables of interest, but the true values for these questions are known to the study team, either at the time of the expert interview or later during the study period. The calibration questions enable empirical validation of the experts’ hypotheses. Experts are scored based on their assessments on the calibration questions, and their assessments on the variables of interest are weighted according to the scores and combined.

Experts’ assessments on the calibration questions are scored in two ways. First, the *statistical accuracy* (also called “calibration”) score reflects how the well an expert’s assessments and the realization data agree. It is based on the Kullback-Leibler divergence measure *I*(*s,p*):

$$I(s,p)=\sum_{i=1}^{6}s_{i}\ln\frac{s_{i}}{p_{i}},$$

where 6 is the number of intervals created by dividing the range of outcomes based on the provided five quantiles, *s*_*i*_ is the observed proportion of the realizations that fall within interval *i*, and *p_i_* = (0.05,0.20,0.25,0.25,0.20,0.05) is the expected proportion of realizations to fall within interval *i*. The statistical accuracy of each expert *e* is then defined as:

$$statistical\;accuracy\;(e)=1-\chi_{5}^{2}(2N\times I(s,p)),$$

where *N* is the number of calibration questions and $\chi_{5}^{2}$ is the cumulative distribution function of a Chi-squared distribution with 5 degrees of freedom. Thus, the statistical accuracy score is the p-value at which the hypothesis that the expert is statistically accurate would be falsely rejected; the scores range from 0 to 1, and higher scores are better.

Second, the *information* score reflects how concentrated or spread out an expert’s distributions are. An expert that provides narrow ranges receives a higher information score than an expert with wide ranges. It is measured as the Shannon relative information with respect to a background measure. As is standard in applications of the Classical Model, we chose a uniform background measure with a 10 percent overshoot, meaning that for each question the minimum and maximum value of the background distribution was determined by taking the smallest interval containing all the expert assessments and the realization (for calibration questions) and extending the interval by plus and minus 10 percent.

The product of statistical accuracy and information is the *combined score*. Performance-weighted combinations are a weighted average of the experts’ assessments, with each expert’s relative weight *w*_*i*_ defined as:

$$w_{i}=statistical\;accuracy\;(e_{i})\times information\;(e_{i})\times cutoff\;indicator{(e}_{i}),$$

where the cutoff indicator equals 1 if the expert’s statistical accuracy score exceeds the cutoff threshold α and equals 0 otherwise. The weights are then normalized to sum to one. The proper scoring rule constraint imposes the use of a cutoff on statistical accuracy, beneath which an expert is unweighted, but does not determine the value of the cutoff. This value is therefore chosen to maximize the combined score of the resulting combination of experts. The resulting weights are asymptotically strictly proper scoring rules, meaning that an expert maximizes her long run expected weight by stating her true beliefs.

More information on the Classical Model’s scoring and weighting mechanisms can be found elsewhere [49,51–53,55,56]. The Classical Model is implemented in the Excalibur software [57].

We identified relevant areas of expertise, including microbiology, epidemiology, public health, and clinical infectious diseases. As we were interested in understanding nationwide trends in resistance rates, an ideal expert would have experience working on antimicrobial resistance at a macro level, rather than only have advanced clinical or laboratory skills. Although antimicrobial resistance has important environmental and veterinary components, we did not recruit experts from these fields as our questions focused solely on human health. We identified experts through our knowledge of relevant clinical and microbiology researchers active in the four countries of interest: France, Italy, Spain, and the United Kingdom. We asked experts to nominate other suitable experts from these countries, who we also contacted and asked to identify additional experts. We repeated this process until no additional new names were provided. We recruited experts from government, health systems, and academia. Although experts from industry would also have knowledge relevant for these questions, they were not included to avoid conflicts of interest.

We conducted remote one-on-one interviews with the experts by web-conference in September–November 2016. Some interviews were two-on-one, with two elicitors and one expert. The elicitor explained the motivation for the study and the use of expert judgment, the use of quantiles to quantify uncertainty, and the scoring mechanisms of the Classical Model. The interview also included three training questions to ensure experts understood how to provide their uncertainty assessments. During the interviews, the elicitor(s) prompted the experts to state the rationale for their assessments, including any data, models, assumptions, and other factors they considered when making judgments, and took notes on their responses. Collecting expert rationales enables the elicitor to check if the quantitative assessments match the qualitative story provided by an expert and can bring to light any differences between how the experts interpret questions so that issues can be clarified. The rationales also help with interpreting and understanding the elicitation results, particularly if there are differences of opinion. The experts were not provided any background information beyond what was contained in the elicitation protocol, and none of the experts reported consulting with data or other sources when explaining how they made their judgements.

The elicitation protocol included 10 calibration questions which drew on data released by the EARS-Net [33,58] and the European Gonococcal Antimicrobial Surveillance Programme (Euro-GASP) [59]. Prior to assessing calibration questions, the protocol introduced potential sources of noise in the calibration data (e.g., the laboratories reporting to EARS-Net may not be consistent from year to year), and experts were instructed to incorporate this uncertainty in their distributions. The protocol had 30 total variables of interest, which concerned future rates of resistance in different pathogen-antibiotic pairs in each of the four countries (France, Italy, Spain, and the United Kingdom). We asked about resistance rates in 2018, 2021, and 2026 for following pathogen-antibiotic pairs, focusing on resistance in invasive isolates:

1. *E*. *coli* and fluoroquinolones
2. *E*. *coli* and third-generation cephalosporins
3. *E*. *coli* and carbapenems
4. *K*. *pneumoniae* and third-generation cephalosporins
5. *K*. *pneumoniae* and carbapenems
6. *S*. *aureus* and methicillin (MRSA)
7. *Streptococcus pneumoniae* and penicillins
8. *Neisseria gonorrhoeae* and third-generation cephalosporins
9. *Pseudomonas aeruginosa* and any available treatment

We also asked experts about resistance rates in 2021 for select non-invasive isolates.

The protocols reported and elicited country-specific resistance rates but were otherwise identical across countries. For each item, we asked experts to provide five values: the 5th, 25th, 50th, 75th, and 95th percentiles from their uncertainty distributions. The 50th percentile was the expert’s median assessment for the item; the expert believed it was equally likely that the true value for the question falls above or below that value. The values for an expert’s 5th and 95th percentiles form a 90% credible range; the expert believed there was a 90% chance the true answer fell between those two values. Similarly, the 25th and 50th percentiles form a 50% credible range. In this way, an expert’s assessments create a statistical hypothesis that can be evaluated against realized data.

The full elicitation protocols and elicited data, including anonymized individual expert responses, are available in in a university-maintained, accessible data repository [60].

### Statistical forecasting

We tested several exponential smoothing and autoregressive integrated moving average (ARIMA) models on the EARS-Net data. Our objective was to compare their outcomes with the expert elicitation outcomes, and not necessarily to find the best fit model. The EARS-Net time-series data includes the percent of tested pathogen-antibiotic pairs that were resistant for each of the countries from 2000 to 2015; the first year of testing varies by country and pathogen-antibiotic pair from 2000 to 2007. Since the data is in the format of a ratio (the number of isolates resistant / the number of isolates tested) with zero values we applied an adjusted logit transformation using the *car* package in R [61], mapping values to the interval (0+*ϵ*,1−*ϵ*). We then fit models to the time-series and forecast the future rates and 50% and 90% prediction intervals for each pathogen-antibiotic-country triplet described previously. We did the analysis using the R *forecast* and *forecastHybrid* packages [62–64].

For comparability with expert uncertainty intervals, we applied exponential smoothing models using the “ets” function from the *forecast* package, which adds an underlying stochastic state space model. The prediction intervals are the percentiles of simulated sample paths. We explore a number of different exponential smoothing methods: simple exponential smoothing with additive errors, which is suitable if the historical data shows no underlying trend or seasonal patterns; Holt’s linear trend with additive errors, which is more suitable for forecasting when we observe a trend in historical data; and lastly Holt’s linear method with a dampened trend, which has been shown to successfully adjust for over-forecasting by Holt’s linear method when the time-horizon is significant [39]. As our data is annual and we do not observe seasonality, we do not consider models with seasonality. For each model, we also computed the corrected Akaike information criterion (AICc) (as described in [39]). S1 Appendix provides further detail on the exponential smoothing models.

Next, we fit the data to an ARIMA(p,d,q) model:

$$y_{t}^{d}=c+\sum_{i}^{p}\phi_{i}y_{t-i}^{d}+\sum_{i}^{q}\theta_{i}e_{t-i}+e_{t},$$

where $y_{t}^{d}$ is the transformed time series differenced *d* times, *c* is a constant term, *ϕ*_*i*_ is the *i*^th^ autoregressive parameter, θ_*i*_ is the *i*^th^ moving average parameter, and *e*_*t*_ is white noise. We fit the ARIMA parameters using the “auto.arima” function in the *forecast* package, which determines the number of differences, d, based on Kwiatkowski–Phillips–Schmidt–Shin (KPSS) unit root, and minimizes the AICc to determine values for p and q (as described in [39]). The maximum values for p, d, and q were set to 2. The point predictions of an ARIMA model with p, d, q of (0,1,1) without a constant are equivalent to the point predictions from simple exponential smoothing, an ARIMA(0,2,2) without a constant model’s point predictions are equivalent to those from the Holt’s linear trend, and an ARIMA(1,1,2) model’s point predictions are equivalent to those from exponential smoothing with a damped linear trend. After evaluating the results from the automatic procedure and determining if errors deviated from white noise, we adjusted models as necessary for three of the pathogen-antibiotic-country triplets (*K*. *pneumoniae* and carbapenems in France, Italy, and Spain). We conducted additional unit root tests (augmented Dickey Fuller [ADF]), evaluated the autocorrelation (ACF) and partial autocorrelation (PACF) functions, plotted the ACF of the residuals, and conducted Ljung Box tests. We used the identified models to project point estimates and prediction intervals. Lastly, since combining forecasts using different methods often leads to better accuracy [65], we also combined results from the “auto.arima” and “ets” functions using equal weighting [64]. The Supplementary Information provides the mean absolute scaled error (MASE) for each forecasting model considered (Table A in S1 Appendix), which is based on in-sample predictions.

## Results

### Expert scores

Table B in S1 Appendix shows the statistical accuracy and information scores for the experts and two combinations of the experts, called “decision makers.” Two weighting schemes are presented: the equal-weight decision maker (EW) assigns all experts the same weight, and the performance-weight decision maker (PW) assigns experts a constant weight for all questions based on their performance on the calibration questions. We also considered item-specific performance weights, but they are not presented as the results in each country were identical or only nominally different from the PW shown. Expert and decision maker scores are based on the calibration questions; assessments for these items are presented in Figs A-D in S1 Appendix.

The France and Italy panels both had one expert who could be deemed “statistically accurate,” meaning their statistical accuracy scores exceeded 0.05, the traditional p-value cut-off used in hypothesis testing. In France, the expert with the highest statistical accuracy score was also the most informative. None of the Spanish experts were statistically accurate, and the United Kingdom panel had two statistically accurate experts. The PW was both more statistically accurate and more informative than the EW in all four countries, and both PW and EW had acceptable statistical accuracy in France, Italy, and the United Kingdom. In Spain, however, both equally-weighting each the experts and weighting the experts according to performance produced a combination of assessments that did not perform well, as seen by the low statistical accuracy score. In Italy and France only one expert received weight in the PW, which happens in about one-third of Classical Model applications [53,66].

### Variables of interest

**Fig 1:**
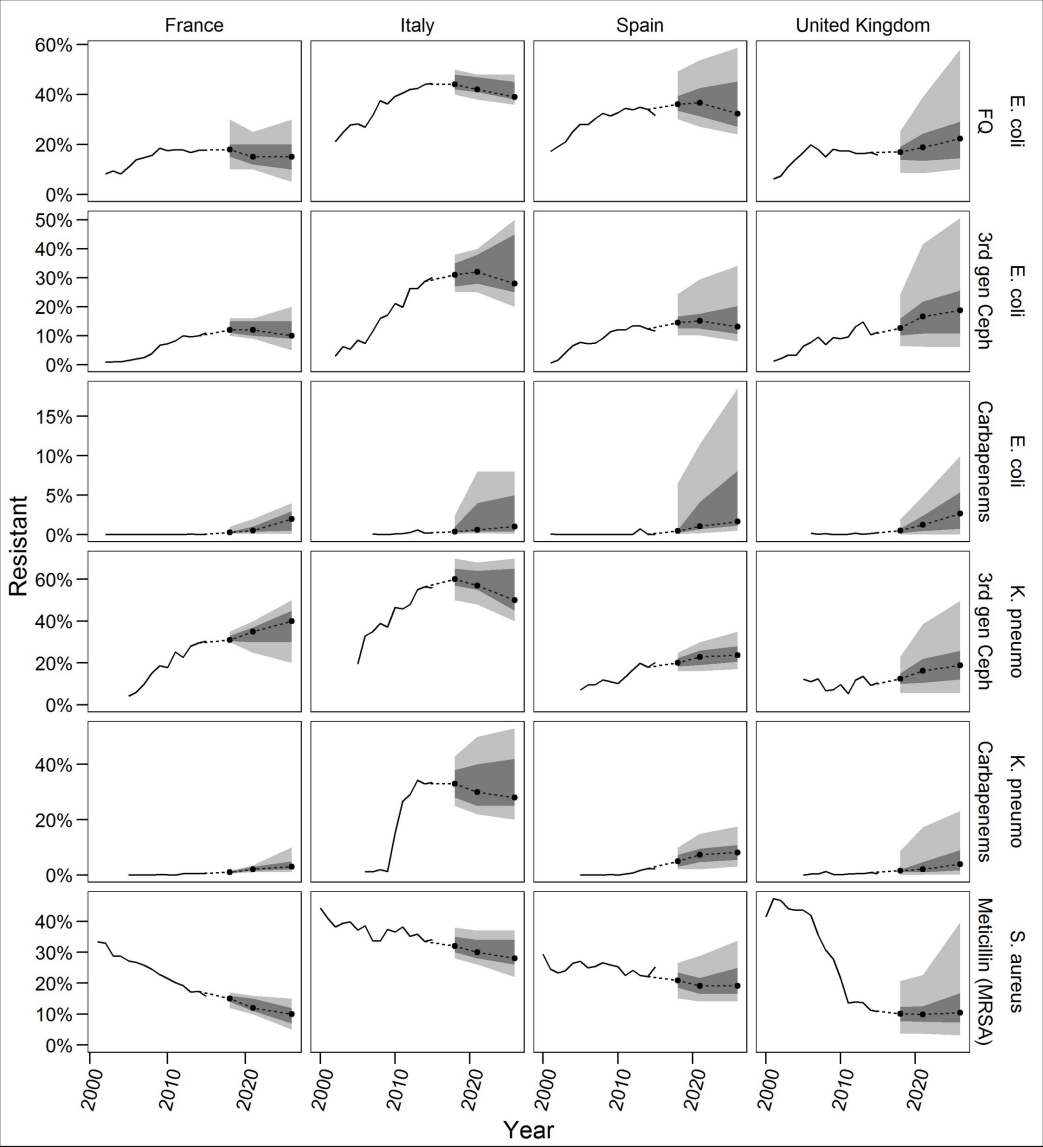
Performance-weight decision maker assessments. Solid lines depict EARS-Net data. Dots indicate the median assessment, dark grey is the 50% credible range, and light grey is the 90% credible range. Experts assessed rates for 2018, 2021, and 2026. Plots linearly extrapolate other future years.

Fig 1 shows the PW assessment for the variables of interest associated with *E*. *coli* resistance to fluoroquinolones, third-generation cephalosporins, and carbapenems; *K*. *pneumoniae* resistance to cephalosporins and carbapenems; and MRSA. Both the experts and historical data, from the European Antimicrobial Resistance Surveillance Network (EARS-Net) only consider invasive isolates [33,58]. Figs E-H in S1 Appendix provide the individual expert assessments for these items, and Figs I-K in S1 Appendix give both the individual expert and decision maker assessments for the additional combinations not discussed here.

The PW’s 90% credible ranges indicate resistance rates for all pathogen-antibiotic pairs in each country could decrease, except when resistance is near zero (e.g., *E*. *coli* and carbapenems in all countries). The PW’s median trajectories show a steady increase in resistance (e.g. *K*. *pneumoniae* and third-generation cephalosporins everywhere but Italy), a steady decline in resistance (e.g., MRSA everywhere except the UK), or a plateau around the current resistance rate, sometimes with a slight near-term increase before coming down (e.g., *E*. *coli* and third-generation cephalosporins everywhere but the UK). The EW’s median estimates are typically similar to the PW’s; the two decision makers’ medians differ by less than five percentage points in 75% of cases. MRSA in France is the only combination with the upper bound of the PW’s 90% credible range falling below the current value. Most of the PW distributions are right-skewed, indicating the possibility of large increases in resistance rates. The PW’s 90% credible ranges are narrower than the EW’s, which tend to be even more right-skewed (Figs A-D in S1 Appendix).

In addition to providing quantitative assessments, experts also gave qualitative information on their rationale behind their judgments. Experts in all countries thought antibiotic stewardship and hospital infection control initiatives would continue and would have an impact on resistance rates for all of the antibiotics discussed. As the use of carbapenems increases, though, experts said *E*. *coli* and *K*. *pneumoniae* resistance to carbapenems would also increase, and many experts talked specifically about the threat of carbapenemases. In Italy, where *K*. *pneumoniae* resistance to carbapenems already exceeds 30% [33], experts thought the attention given to infection control in reaction to the large spike in resistance could make the rate stabilize or decline in the future, but further increases are also possible. All four countries agreed that MRSA control would continue to be effective, so rates would likely decline or improvements in MRSA rates would be sustained in the future. In the United Kingdom, where MRSA rates were near 50% in the early 2000s [33], some experts thought there was a chance rates could rise near that level again if control efforts became lax or a new resistant clonal group emerged.

### Comparison to statistical forecasts

We created a series of exponential smoothing and autoregressive integrated moving average (ARIMA) forecasting models (Figs M-S in S1 Appendix). Here, we present some comparisons illustrating the differences that exist between the various methods.

**Fig 2:**
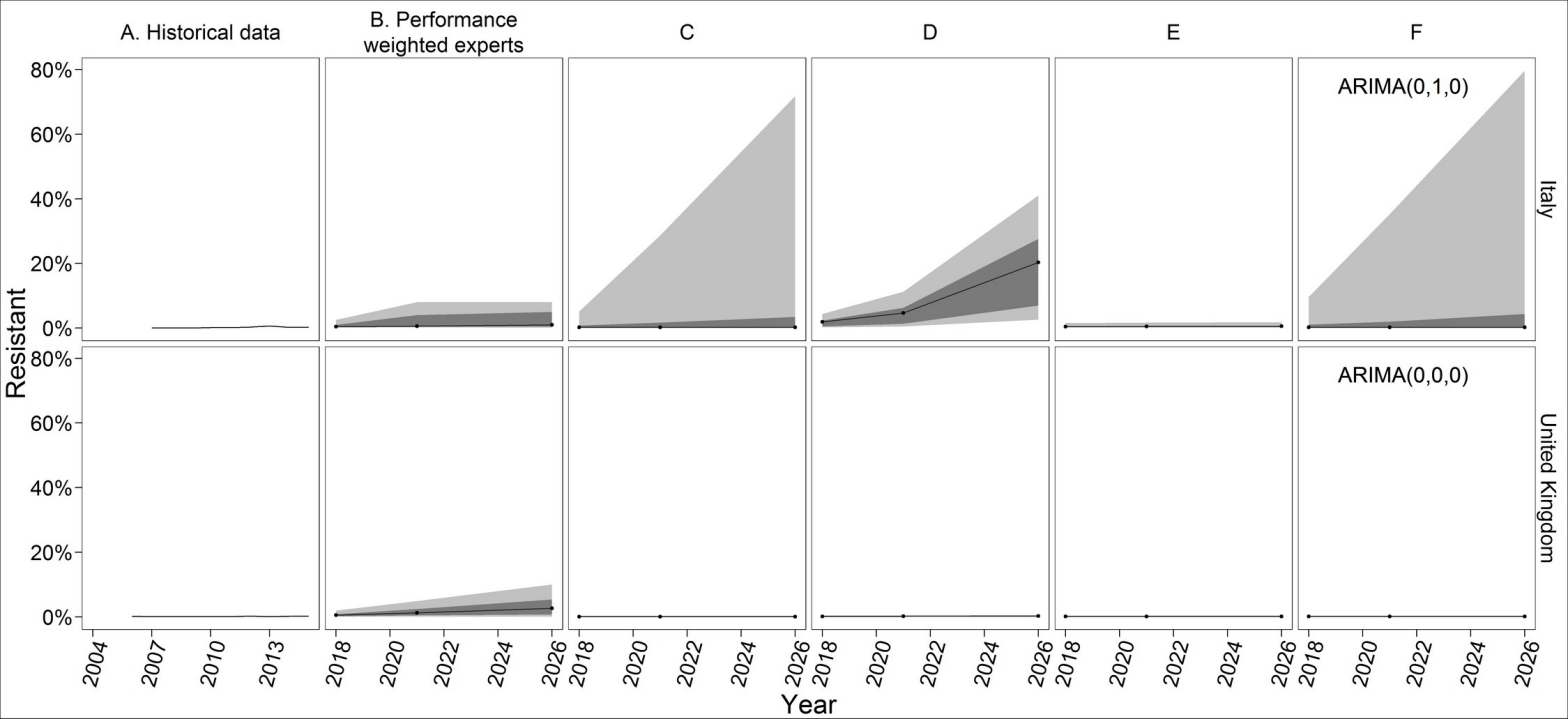
PW and forecasting results for *E*. *coli* resistance to carbapenems in Italy and the United Kingdom. All forecasts begin with 2018, 3 years after the most recent historical data. Black lines indicate the median, dark grey indicates the 50% prediction interval, and light grey indicates the 90% predication interval. The experts only assessed 2018, 2021, and 2026. The ETS models use exponential smoothing with additive error, no seasonality, and either no trend (Panel C), an additive trend (Panel D), or a damped trend (Panel E). ARIMA models (Panel F) are labelled with the ARIMA(p,d,q), values selected for that country-pathogen-antibiotic model, where p = the order of the autoregressive model, d = the degree of differencing, and q = the order of the moving average model.

*E*. *coli* resistance to carbapenems has not yet exceeded 1% in any of the countries [58]. The statistical forecasts in the UK all produce narrow 90% prediction intervals with the upper bound less than 1% (Fig 2). This pattern also holds in France and Spain (Figs M-S in S1 Appendix). Experts, however, thought resistance would slowly increase, and the PW assessments reflect at least a 50% chance of *E*. *coli* resistance to carbapenems exceeding 1% in 2026 (Fig 1). While the statistical models only take into account the low historical rates of resistance, experts considered the increased consumption of carbapenems. However, most experts also thought resistance would not exceed 10% (i.e., the upper bounds of their 90% credible ranges do not exceed 10%), as carbapenems are not used in the community and hospital infection control should contain its spread.

Experts in Italy described a similar story, and observed rates of *E*. *coli* resistance to carbapenems in Italy have also remained below 1%. However, the pattern and variance of the EARS-Net observations is different, Italy observed two years with a resistance rate of 0%, and the statistical forecasts vary (Fig 2). The upper bounds of the exponential smoothing model with no additive trend and the ARIMA model (Fig 2, Panels C and F) are much higher than that of the PW combination, the other statistical forecasts, or any of the individual experts (Fig F in S1 Appendix). The additive trend model is the only forecast with a median projection in 2026 greater than 1% (Fig 2, Panel D). The upper bound of the damped trend model is 1.9% (Fig 2, Panel E), much lower than the upper bound from any of the other forecasts.

**Fig 3:**
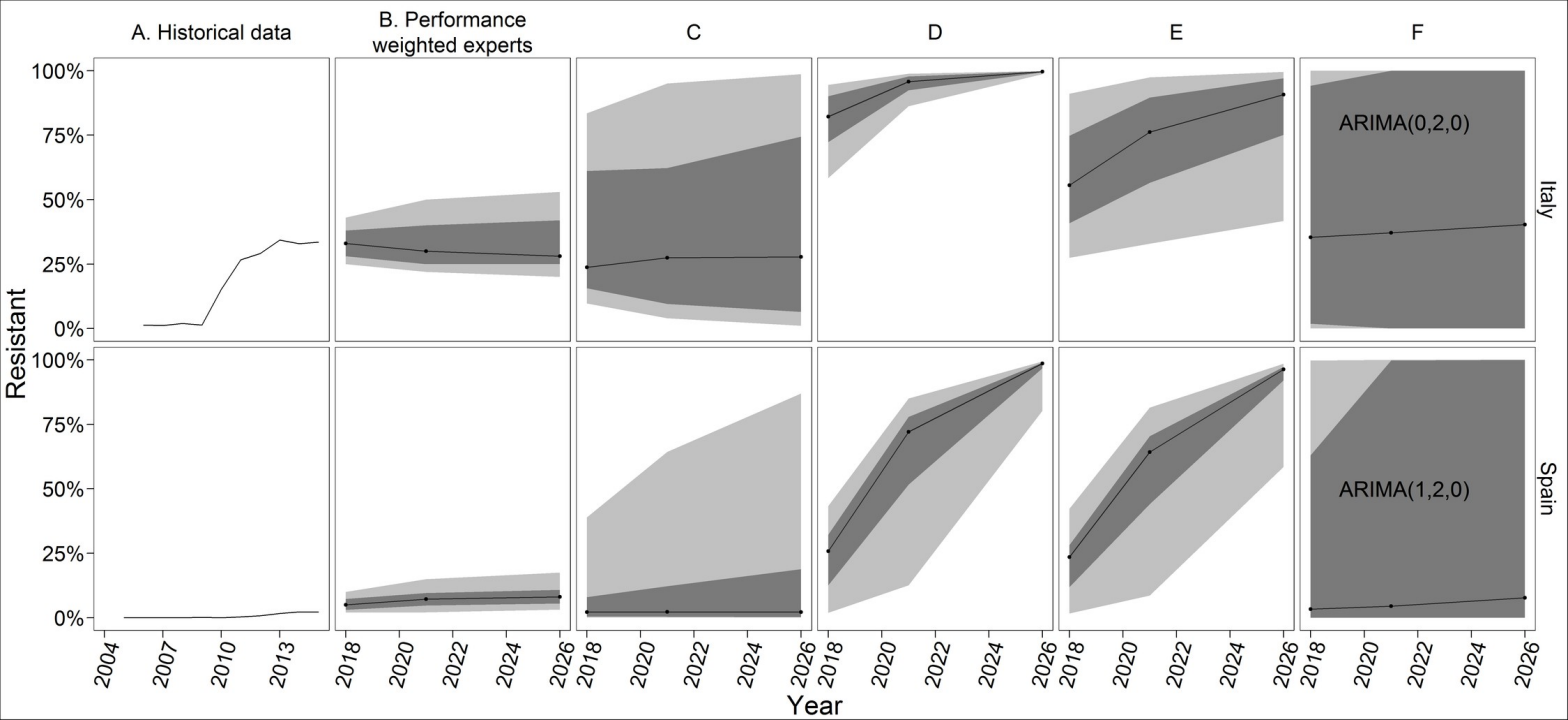
PW and forecasting results for *K*. *pneumoniae* resistance to carbapenems in Italy and Spain. All forecasts begin with 2018, 3 years after the most recent historical data. Black lines indicate the median, dark grey indicates the 50% prediction interval, and light grey indicates the 90% predication interval. The experts only assessed 2018, 2021, and 2026.

The proportion of K. pneumoniae isolates resistant to carbapenems in Italy rose from 1.3% in 2009 to 26.7% in 2011 [33]. Experts thought the attention given to infection control in reaction to the large spike in resistance could make the rate stabilize, but further increases are possible and regional variation within the country increased uncertainty around the median (Fig 3). The exponential smoothing model with no trend and the ARIMA model both produce median estimates similar to the experts, but with much more uncertainty. The exponential smoothing model’s 90% prediction interval ranges from 1% to 99% (Fig 3, Panel C), and the ARIMA model’s 50% prediction interval ranges from 0% to 100% (Fig 3, Panel F). Both models give a high likelihood of extreme scenarios. The additive trend model (Fig 3, Panel D), however, projects that near-complete *K*. *pneumoniae* resistance to carbapenems in Italy is almost certain, with the 90% prediction interval in 2026 ranging from 98.7% to 99.9%. Damping the trend (Fig 3, Panel E) decreases the median and widens the prediction interval relative to the non-damped additive trend, but the median projection in 2026 is still higher than the experts or the other two statistical forecasts.

Experts in the other three countries thought *K*. *pneumoniae* resistance to carbapenems would follow a similar pattern as *E*. *coli* resistance, but with a higher ceiling, reflecting that *K*. *pneumoniae* is harder to remove from the environment, resistance genes spread more quickly among *K*. *pneumoniae*, and Italy experienced a sharp increase in resistance for this pathogen-antibiotic pair, demonstrating a rapid change is possible. The statistical forecast models in Spain, however, all overestimate the future resistance and/or the uncertainty about future resistance relative to the experts (Fig 3). As the additive trend model (Fig 3, Panel D) identifies a linear trend in the data and cannot increase above 100%, the model indicates increasing certainty into the future. The ARIMA model has extremely wide prediction intervals, reflecting high variability in errors that, due to the second-order differencing, are based on only a few observations.

**Fig 4:**
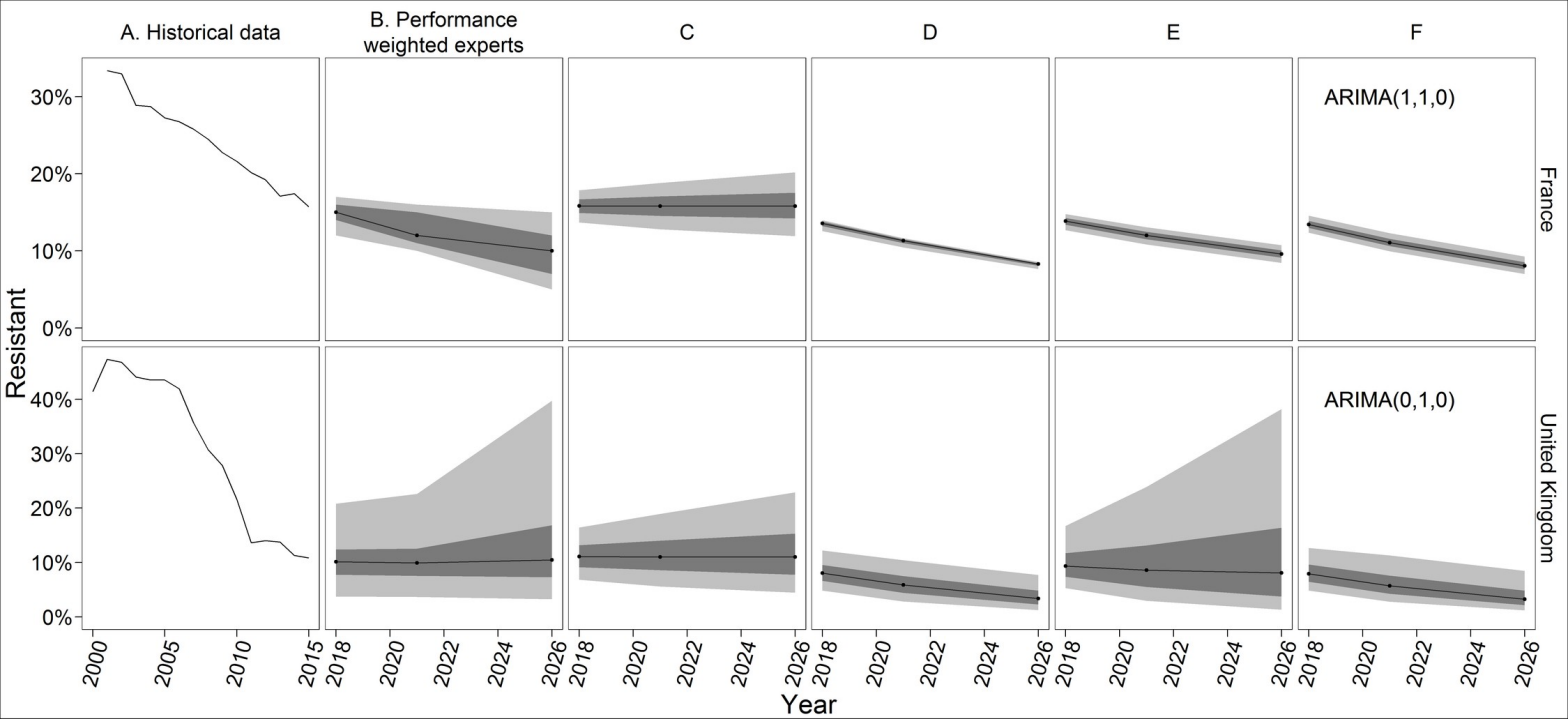
PW and forecasting results for MRSA in France and United Kingdom. All forecasts begin with 2018, 3 years after the most recent historical data. Black lines indicate the median, dark grey indicates the 50% prediction interval, and light grey indicates the 90% predication interval. The experts only assessed 2018, 2021, and 2026.

Experts everywhere thought MRSA control would continue to be effective, but in some countries experts also said that MRSA rates could hit a floor and not fall further. This logic and the corresponding PW estimates align with some of the statistical forecasts. The median of the PW in France, for example, is similar to the median projections of three of the four statistical forecast models (Fig 4), though the models’ 90% prediction intervals are narrower than the PW’s 90% credible range. In the UK, the PW estimates and credible ranges are similar to the exponential smoothing model with a damped trend (Fig 4, Panel E). In this case, the intuition of the model matches the logic of the experts, who thought resistance would decline at a decreasing rate as it approaches a floor (i.e., a damped trend). The upper bound of the UK PW’s credible range reflects that some experts thought the MRSA rate could approach 50%, as it did in the early 2000s 30, if control weakened or a new clonal group emerged. This is coincidentally similar to the upper bound of the damped trend model’s prediction interval. However, the exponential smoothing model with no trend has a better fit to the historical data, as determined by minimizing the corrected Akaike’s Information Criterion (AICc), a standard approach for choosing among exponential smoothing or ARIMA models.

## Discussion

In this paper, we present estimates and uncertainty ranges—from judgmental forecasts and statistical forecasts—for the future trajectory of resistance for six pathogen-antibiotic pairs. Four of these pairs—*E*. *coli* and *K*. *pneumoniae* resistance to third-generation cephalosporins and carbapenems—are critical public health priorities according to the World Health Organization’s (WHO) 2017 priority pathogens list [67], and MRSA is a high priority. Experts in France, Spain, and the United Kingdom thought resistance for all of these priority pathogens would remain below 50% in 2026, according to the PW’s 90% credible ranges. The EW’s 90% credible ranges are wider, but its 50% credible ranges also stay below 50% resistance for all of these combinations except fluoroquinolone-resistant *E*. *coli* in Spain in 2026, when the upper bound of the 50% credible range is 52.5% resistance. In Italy, the PW’s 95^th^ percentile estimates reach or exceed 50% resistance for *E*. *coli* and third-generation cephalosporins, *K*. *pneumoniae* and third-generation cephalosporins, and *K*. *pneumoniae* and carbapenems, but the medians suggest Italy will sustain or improve its current rates for these pairs.

The experts’ judgments were based on their belief that the increasing local, national, and global focus on antibiotic stewardship in hospitals and the community and hospital infection control will continue and will positively impact resistance rates. These results do not mean antibiotic resistance control efforts can be relaxed, but that experts believe they have had and will continue to have a positive impact on resistance rates. The distributions from the experts are conditional on their expectations that these programs will continue or expand and do not represent what would happen if the programs ended, although some experts discussed this possibility when determining the upper bound of their 90% credible interval. Although we did not ask about future resistance in a specific scenario with decreasing or no antibiotic stewardship and infection control, many of the experts mentioned these programs were a key factor in their estimates, underscoring their importance for preserving antibiotic effectiveness. Future work could ask experts about the trajectory of resistance under different policy or intervention scenarios, to get more information on the impact of specific policies.

The experts’ assessments also indicate the role antibiotic resistance surveillance programs play in preserving antibiotic effectiveness. Experts who discussed a possible future increase in MRSA mentioned a scenario in which a new clonal resistant strain emerges and establishes quickly, undoing previous improvements in the rate of MRSA. Effective surveillance systems with timely information sharing are needed to identify and contain new resistant strains quickly after they emerge to prevent this.

Our study includes three of the six pathogens included in the AMR Review report on the burden of antimicrobial resistance (the AMR Review also included HIV, tuberculosis, and malaria) [68]. The AMR Review estimates burden building on two commissioned reports that make assumptions about future rates of resistance through 2050. The RAND Europe report includes resistance changing to 5%, 40%, and 100% across all countries and pathogens [69], and the KPMG report considers increasing resistance to 40% higher than reported 2011 rates or to 100% [70]. Although our projections only go through 2026–10 years from the time of the interviews—the AMR Review scenarios are not consistent with the findings presented here.

We show two approaches to forecasting, a purely judgment-based method and a suite of statistical approaches. The statistical methods make most explicit use of the historical data, interpreted through specific underlying mathematical modelling, but are not able to anticipate changes in antibiotic use and infection control, the emergence of new resistant strains, or the introduction of new antibiotics. Experts have relevant knowledge about these issues and their impact on future resistance rates, which should be included in forecasts. Expert forecasts can be limited by heuristics and biases, but structured elicitation approaches, such as the Classical Model, are a way to minimize their impact on results [41].

We present these as separate methods, but judgmental and statistical forecasts can also be combined in different ways [37,71]. Results from the two approaches can be averaged, for example either with equal weight given to the experts and statistical forecast or using the Classical Model’s performance weights and producing distributions for the calibration questions from the statistical forecasts. This does not resolve the choice of which statistical forecast to use, however, as different model structures can produce different results, as seen here.

An expert, rather than AICc or another measure of model fit, can choose the modelling approach and structure (e.g., selecting an exponential smoothing model with a damped trend for some pathogen-antibiotic pairs and a model with no trend for others). Experts can also introduce additional model parameters to make statistical forecasts better reflect what they anticipate will happen (e.g., the idea that in practice resistance wouldn’t rise above some threshold, see Fig R in S1 Appendix). Making these judgments requires experts who have both subject-matter expertise relevant for understanding future resistance rates and technical expertise enabling informed choice on model structure and parameters. This introduces additional cognitive burden on the experts and may be a less natural way for them to think about future resistance rates.

Another method to combine approaches is to use the statistical forecasts as an “input” in judgmental forecasts, as background material given to the experts. If this is done, however, care must be given in the elicitation to discourage experts from anchoring on the historical trend and inadequately adjusting their assessments (anchoring and adjustment are discussed in [40]). The problem could possibly be minimized by sharing a range of forecasts and discussing the likelihood of the various results and the factors contributing to different scenarios. Asking the experts for the rationale for their judgments and challenging any assumptions they make, best practice in any elicitation, could also help reduce bias if statistical forecasts were used in this way.

If experts anticipate recent and future events are more important than characterizing past trends for forecasting the future, statistical forecasting might not improve judgmental forecasts. Conversely, for outcomes for which the past is a good predictor of the future, expert judgment would not have additional value relative to statistical forecasting. In this study, experts demonstrated relevant knowledge about future rates of resistance that was not captured in the statistical forecasts, indicating the potential of expert judgment in this application.

Our study has several important limitations. The resistance rate forecasts presented only apply to invasive isolates; neither the experts nor the statistical forecasts consider resistance rates in hospital- or community-acquired urinary tract infections, skin and soft tissue infections, or other non-invasive infections. Resistance rates in non-invasive isolates may differ from rates in invasive isolates, limiting the interpretation of our results. We did ask the experts three questions about resistance rates for certain non-invasive isolates at one point of time (2021), and these results are in the Supplemental Information (Fig L in S1 Appendix), but eliciting comparisons of invasive versus non-invasive isolates for all relevant combinations and timeframes was not practical. Also, we consider only the rate of resistance, not the number of resistant infections. Resistance rates are an important indicator to inform decisions about control strategies or the need for new drugs, but they do not tell the complete story, and information about the incidence of resistant infections is also needed to guide policy and research [72,73].

The results from our experts indicate most of the antibiotics studied will likely remain effective in the next decade for treating the majority of infections caused by the pathogens discussed in France, Italy, Spain, and the United Kingdom. This result may not hold for all countries, especially mid-income countries like Brazil, India, and China which have recently seen the largest increases in antibiotic consumption [74]. We considered the rate of resistance for each pathogen-antibiotic pair at each point of time to be independent. However, experts discussed dependencies between these rates. Rising resistance to third-generation cephalosporins, for example, could lead to increased use of carbapenems and thus higher carbapenem-resistance rates in the future. We may underestimate the probability of increased resistance if we ignore this dependency. Future work is needed to better understand the magnitude of this issues and its implications for managing the risks of antibiotic resistance.

The judgmental forecasts presented here reflect the experts’ estimation of the uncertainty about future resistance rates in autumn 2016, when the elicitations were conducted. However, the experts’ understanding is constantly evolving as antibiotic resistance surveillance programs continue and expand, new research improves knowledge about the dynamics of resistance, and relevant polices change. Future iterations of this work are needed to allow experts to update their forecasts to reflect this changing information. Researchers conducted an expert elicitation exercise predicting risks from volcanic activity on the island of Montserrat for 14 years [46,48]. A similar ongoing elicitation exercise could be conducted on future rates of resistance, with experts regularly updating their quantitative assessments to reflect the changing landscape of resistance, providing up-to-date information to decision makers. Future work should also evaluate the accuracy of these expert and statistical projections once resistance data from 2018 becomes available.

## Supporting information

S1 Appendix Additional methods and results. (PDF)
