# Supplementary material for: Quantifying uncertainty about future antimicrobial resistance: Comparing structured expert judgment and statistical forecasting methods
Source: PLoS One. 2019 Jul 5;14(7):e0219190. doi: 10.1371/journal.pone.0219190 (PMC6611586; doi:10.1371/journal.pone.0219190)
Supplement: S1 Appendix — (PDF) [file pone.0219190.s001.pdf]

Additional methods

Exponential smoothing attaches greater weights to more recent observations. Here we describe each of the exponential smoothing methods we apply. Please note that the notation is the same as in [1].

Simple exponential smoothing is a weighted average of all historical observations as follows (equations from [1]):

$$\hat{y}_{t+h|t} = l_t$$

$$l_t = \alpha y_t + (1 - \alpha) l_{t-1}$$

where l_t is the estimate for the level at time t and $0 \leq \alpha \leq 1$ is the level smoothing parameter.

In Holt's linear trend method, we weight both the level and the trend as follows (equations from [1]):

$$\hat{y}_{t+h|t} = l_t + h b_t$$

$$l_t = \alpha y_t + (1 - \alpha)(l_{t-1} + b_{t-1})$$

$$b_t = \beta (l_t - l_{t-1}) + (1 - \beta) b_{t-1}$$

where l_t is the estimate for the level at time t, $0 \leq \alpha \leq 1$ is the level smoothing parameter, and b_t is the estimate for the trend at time t, $0 \leq \beta \leq 1$ is the trend smoothing parameter.

Lastly, we introduce a dampened trend in Holt's linear method as follows (equations from [1]):

$$\hat{y}_{t+h|t} = l_t + (\theta + \cdots + \theta^h) b_t$$

$$l_t = \alpha y_t + (1 - \alpha)(l_{t-1} + \theta b_{t-1})$$

$$b_t = \beta(l_t - l_{t-1}) + (1-\beta)\theta b_{t-1}$$

where $0 < \theta < 1$ is the dampening parameter.

The exponential smoothing methods provide point estimate forecasts. For further detail on the projection intervals and innovations state space models please see [1] Chapter 7.5.

Additional results

Mean absolute scaled error

Table A presents the mean absolute scaled error (MASE) for each of the statistical forecasting models. MASE is based on the model's point predictions. $MASE = mean(|q_J|)$ where q_j is the error scaled by the naïve forecast, such that:

$$q_j = \frac{e_j}{\frac{1}{T-1}\sum_{t=2}^{T} |y_t - y_{t-1}|}.$$

A lower MASE indicates better performance. There is no clear pattern for model performance across the different country-pathogen-antibiotic combinations.

Table A. Mean absolute scaled error (MASE) of each statistical forecasting model.

Country	Bug	Drug	ETS(ANN)	ETS(AAN)	ETS(AA$_d$N)	ETS(ZZZ)	ARIMA		Hybrid	ARIMA bounded	
France	E. coli	FQ	0.9286	0.875	0.7479	0.9286	ARIMA(0,1,0)	0.8499	0.866	ARIMA(0,1,0)	0.8436
		3rd gen Ceph	0.9286	0.6248	0.5636	0.6248	ARIMA(0,1,0)	0.6264	0.6256	ARIMA(0,1,0)	0.6177
		Carbapenems	0.6615	0.5793	0.6342	0.6615	ARIMA(1,1,0)	0.5514	0.5602	ARIMA(1,1,0)	0.5514
	K. pneumo	3rd gen Ceph	0.9091	0.533	0.3164	0.9091	ARIMA(0,1,0)	0.6744	0.7512	ARIMA(0,1,0)	0.6601
		Carbapenems	0.8395	0.6118	0.6854	0.8395	ARIMA(0,0,0)	0.741	0.784	ARIMA(0,0,0)	0.7538
	S. aureus	Meticillin (MRSA)	0.9334	0.3958	0.4899	0.3958	ARIMA(1,1,0)	0.4233	0.3546	ARIMA(1,1,0)	0.4383
Italy	E. coli	FQ	0.9535	0.721	0.4947	0.4947	ARIMA(0,1,0)	0.7799	0.5583	ARIMA(0,1,0)	0.7013
		3rd gen Ceph	0.9317	0.6081	0.5913	0.6081	ARIMA(1,1,0)	0.4886	0.4932	ARIMA(1,1,0)	0.4228
		Carbapenems	0.889	0.9686	0.8387	0.889	ARIMA(0,1,0)	0.8897	0.8893	ARIMA(0,1,0)	0.8896
	K. pneumo	3rd gen Ceph	0.9092	0.6471	0.6599	0.9092	ARIMA(0,1,0)	0.8509	0.7807	ARIMA(0,1,0)	0.7818
		Carbapenems	0.9	1.1871	1.0642	0.9	ARIMA(0,1,0)	1.4144	0.9004	ARIMA(0,1,0)	0.9006
	S. aureus	Meticillin (MRSA)	0.8842	0.8752	0.732	0.8842	ARIMA(0,1,0)	0.9377	0.897	ARIMA(0,1,0)	0.938
Spain	E. coli	FQ	0.9334	0.656	0.4829	0.4829	ARIMA(2,2,2)	0.2842	0.3122	ARIMA(0,2,1)	0.6683
		3rd gen Ceph	0.9341	0.5716	0.556	0.5716	ARIMA(0,2,0)	0.568	0.5675	ARIMA(0,2,0)	0.5787
		Carbapenems	0.9705	0.9301	1.1177	0.9705	ARIMA(0,0,0)	0.9705	0.9705	ARIMA(0,0,0)	0.9703
	K. pneumo	3rd gen Ceph	0.9091	0.544	0.5414	0.9091	ARIMA(0,1,0)	0.764	0.825	ARIMA(0,1,0)	0.7651
		Carbapenems	0.9436	0.6945	0.7379	0.9436	ARIMA(0,1,0)	1.1259	0.922	ARIMA(0,1,0)	0.9098
	S. aureus	Meticillin (MRSA)	0.9663	0.8722	0.895	0.9663	ARIMA(0,0,0)	0.9007	0.9106	ARIMA(0,0,0)	0.9001
United Kingdom	E. coli	FQ	0.9334	0.776	0.6984	0.9334	ARIMA(0,2,1)	0.7656	0.7428	ARIMA(0,2,1)	0.7527
		3rd gen Ceph	0.9334	0.774	0.7203	0.9334	ARIMA(0,1,0)	0.789	0.8182	ARIMA(0,1,0)	0.7827
		Carbapenems	0.7365	0.7124	0.7687	0.7365	ARIMA(0,0,0)	0.7365	0.7365	ARIMA(0,0,0)	0.7365
	K. pneumo	3rd gen Ceph	0.7198	0.7161	0.7002	0.7198	ARIMA(0,0,0)	0.7198	0.7198	ARIMA(0,0,0)	0.7205
		Carbapenems	0.8712	0.7753	0.7414	0.8712	ARIMA(0,0,0)	0.8708	0.871	ARIMA(0,0,0)	0.8699
	S. aureus	Meticillin (MRSA)	0.9375	0.848	0.8644	0.9375	ARIMA(0,1,0)	0.8466	0.8229	ARIMA(0,1,0)	0.8106

Expert and decision maker scores

Table B. Scores and weights of experts, the equal-weight decision maker (EW), and the performance-weight decision maker (PW). Experts with non-zero weight in the PW are bolded.

Expert ID	Statistical accuracy	Information score		Combined score	Weight in PW
		All variables	Calibration variables		
**France**					
1	2.20E-04	1.796	1.471	3.24E-04	0
2	0.031	1.176	1.379	0.043	0
3	1.99E-07	0.706	0.716	1.43E-07	0
4	0.002	0.665	0.671	0.001	0
**5**	**0.652**	**1.528**	**1.958**	**1.276**	**1**
PW	0.652	1.528	1.958	1.276	
EW	0.078	0.519	0.433	0.034	
**Italy**					
1	0.027	0.9	0.632	0.017	0
2	0.017	0.456	0.461	0.008	0
**3**	**0.447**	**0.676**	**0.466**	**0.209**	**1**
4	5.56E-06	0.987	0.99	5.50E-06	0
PW	0.447	0.676	0.466	0.209	
EW	0.218	0.198	0.197	0.043	
**Spain**					
**1**	**1.22E-05**	**0.958**	**0.572**	**6.98E-06**	**0.23**
2	1.03E-09	1.486	1.452	1.49E-09	0
3	1.99E-07	0.258	0.424	8.43E-08	0
4	3.23E-07	1.508	1.642	5.31E-07	0
**5**	**2.24E-05**	**1.378**	**1.043**	**2.33E-05**	**0.77**
PW	3.59E-05	0.869	0.667	2.39E-05	
EW	1.22E-05	0.366	0.231	2.82E-06	
**United Kingdom**					
1	0.002	0.399	0.473	0.001	0
**2**	**0.016**	**1.161**	**1.828**	**0.028**	**0.091**
**3**	**0.181**	**1.096**	**1.13**	**0.205**	**0.658**
**4**	**0.185**	**1.043**	**0.393**	**0.073**	**0.234**
**5**	**0.003**	**1.856**	**1.99**	**0.005**	**0.017**
6	1.96E-08	1.769	0.787	1.54E-08	0
PW	0.499	0.725	0.606	0.302	
EW	0.132	0.532	0.331	0.044	

Expert and decision maker assessments on calibration questions

Fig A-D show the individual expert assessments and decision maker assessments for the 10 calibration questions in each country. Calibration questions were based on data from the European Antimicrobial Resistance Surveillance Network (EARS-Net) [2,3] and the European Gonococcal Antimicrobial Surveillance Programme (Euro-GASP) [4]. The full question text and expert data can be found in the elicitation protocol, available in the University of Strathclyde's PURE data repository at http://dx.doi.org/10.15129/953210ee-27c0-4042-8fd6-f1c5b7325eae.

Fig A. France: Expert, equal-weight decision maker (EW), and performance-weight decision maker (PW) assessments for calibration questions. Boxplots show the median estimate, 50% credible range, and 90% credible range. The dotted line is the realization for that item.

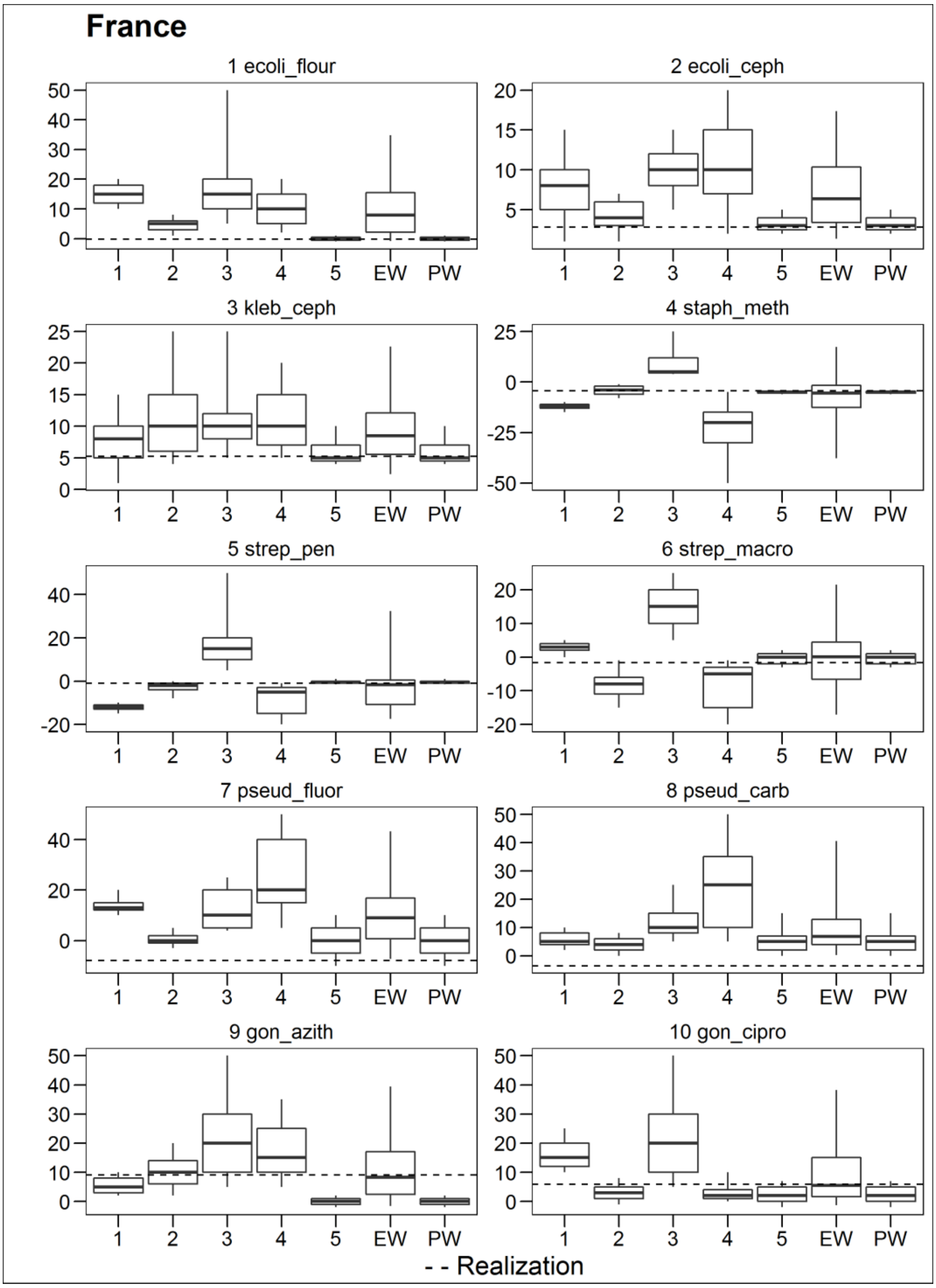

Fig B. Italy: Expert, equal-weight decision maker (EW), and performance-weight decision maker (PW) assessments for calibration questions. Boxplots show the median estimate, 50% credible range, and 90% credible range. The dotted line is the realization for that item.

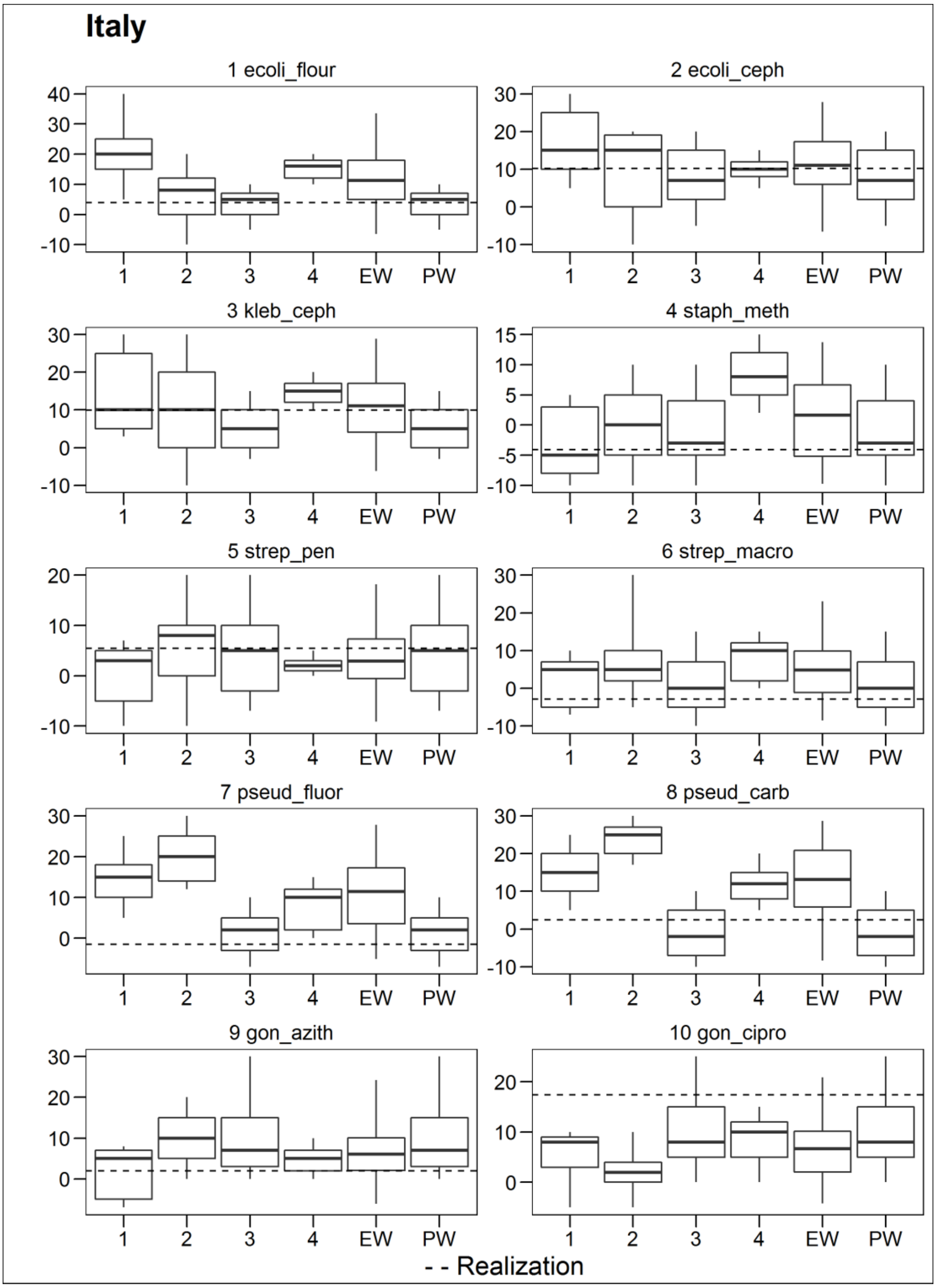

Fig C. Spain: Expert, equal-weight decision maker (EW), and performance-weight decision maker (PW) assessments for calibration questions. Boxplots show the median estimate, 50% credible range, and 90% credible range. The dotted line is the realization for that item.

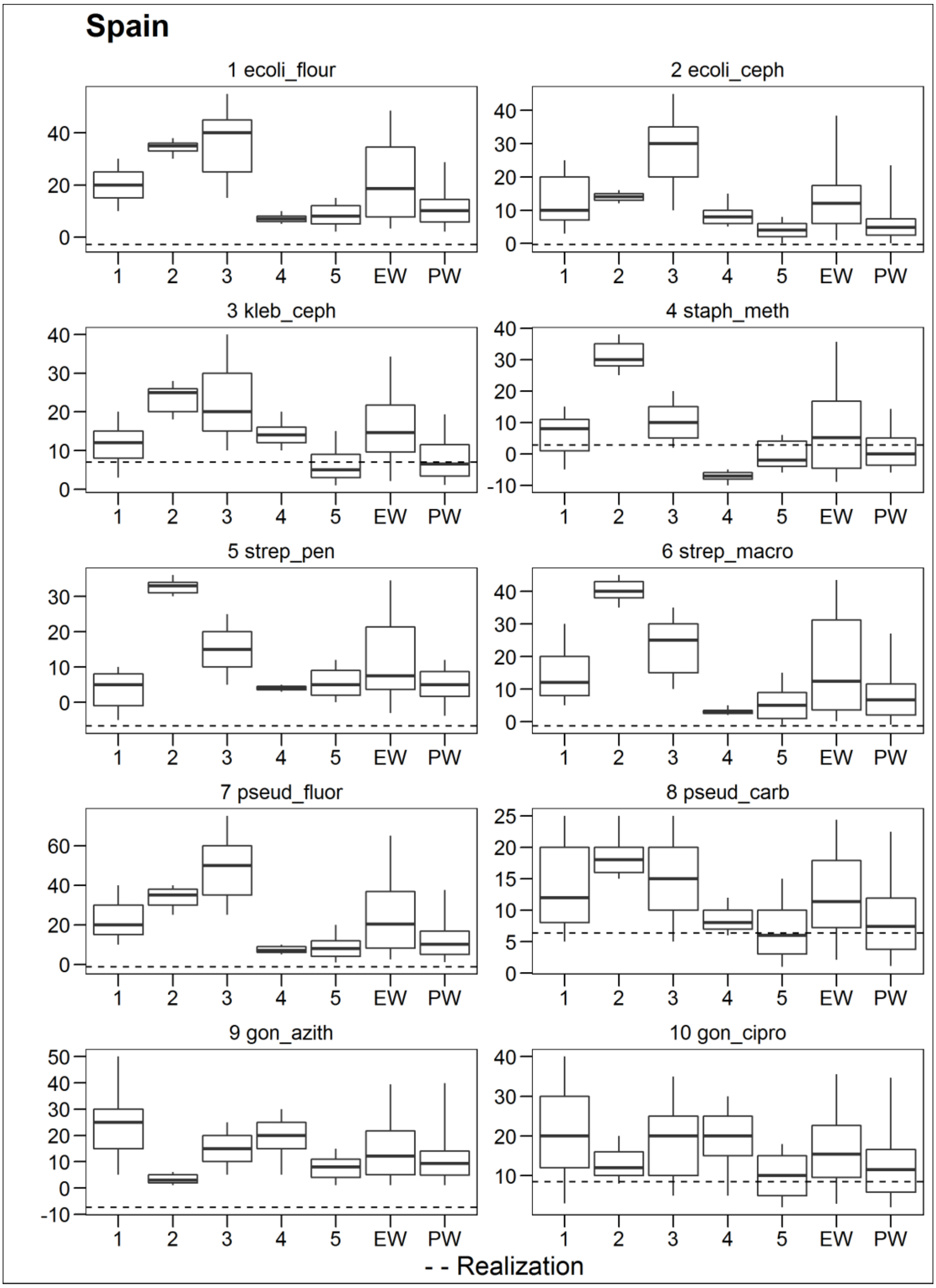

Fig D. United Kingdom: Expert, equal-weight decision maker (EW), and performance-weight decision maker (PW) assessments for calibration questions. Boxplots show the median estimate, 50% credible range, and 90% credible range. The dotted line is the realization for that item.

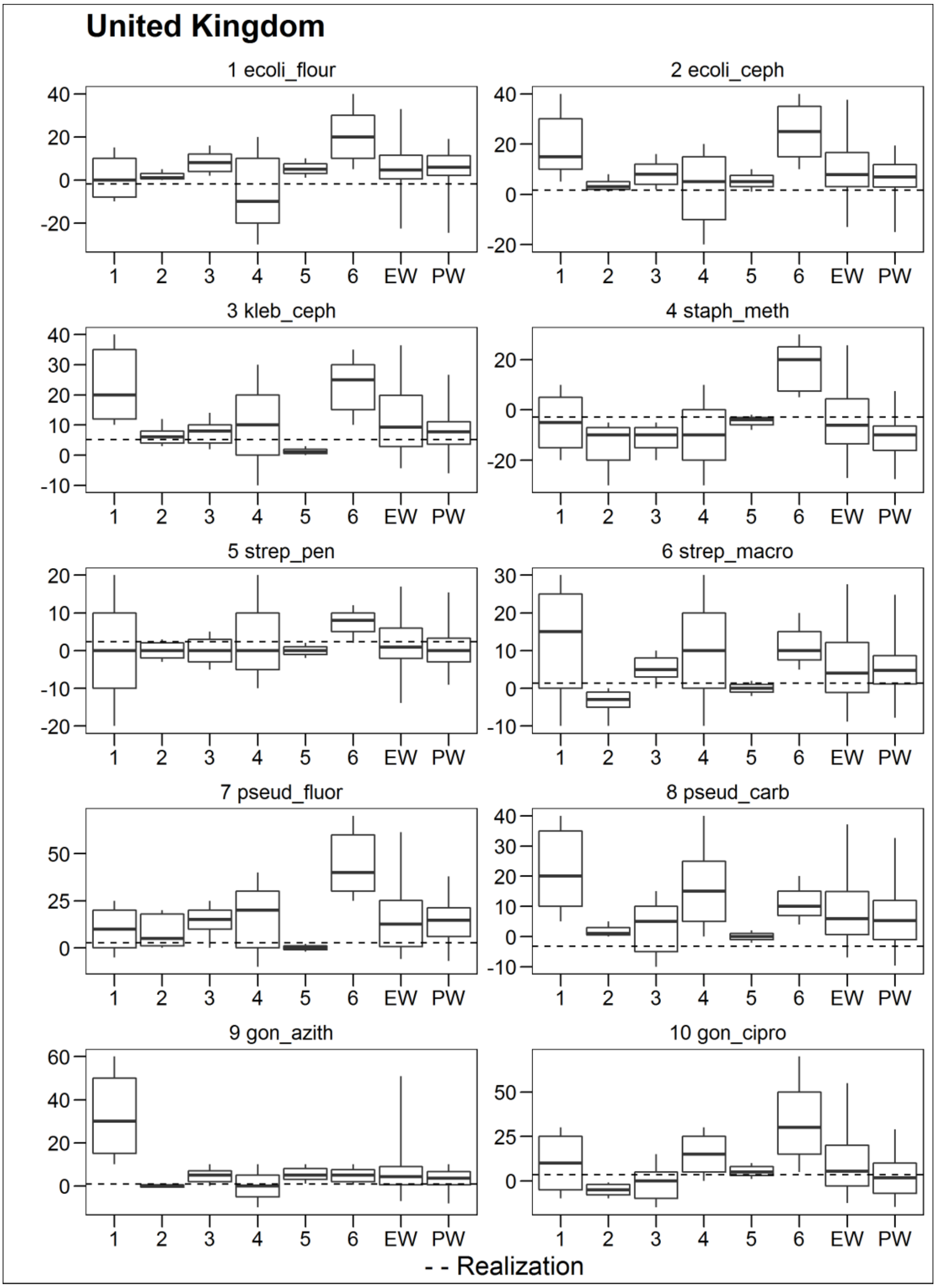

Expert assessments for the variables of interest

Figs E-H show the individual expert assessments and both the equal-weight and performance-weight decision maker assessments for the variables of interest in each country. Figs I-K show results for three pathogen-antibiotic pairs not discussed in the paper, and Fig L provides results for a set of questions about non-invasive isolates.

Fig E. France: Expert, equal-weight decision maker (EW), and performance-weight decision maker (PW) assessments for the variables of interest. Boxplots show the median estimate, 50% credible range, and 90% credible range.

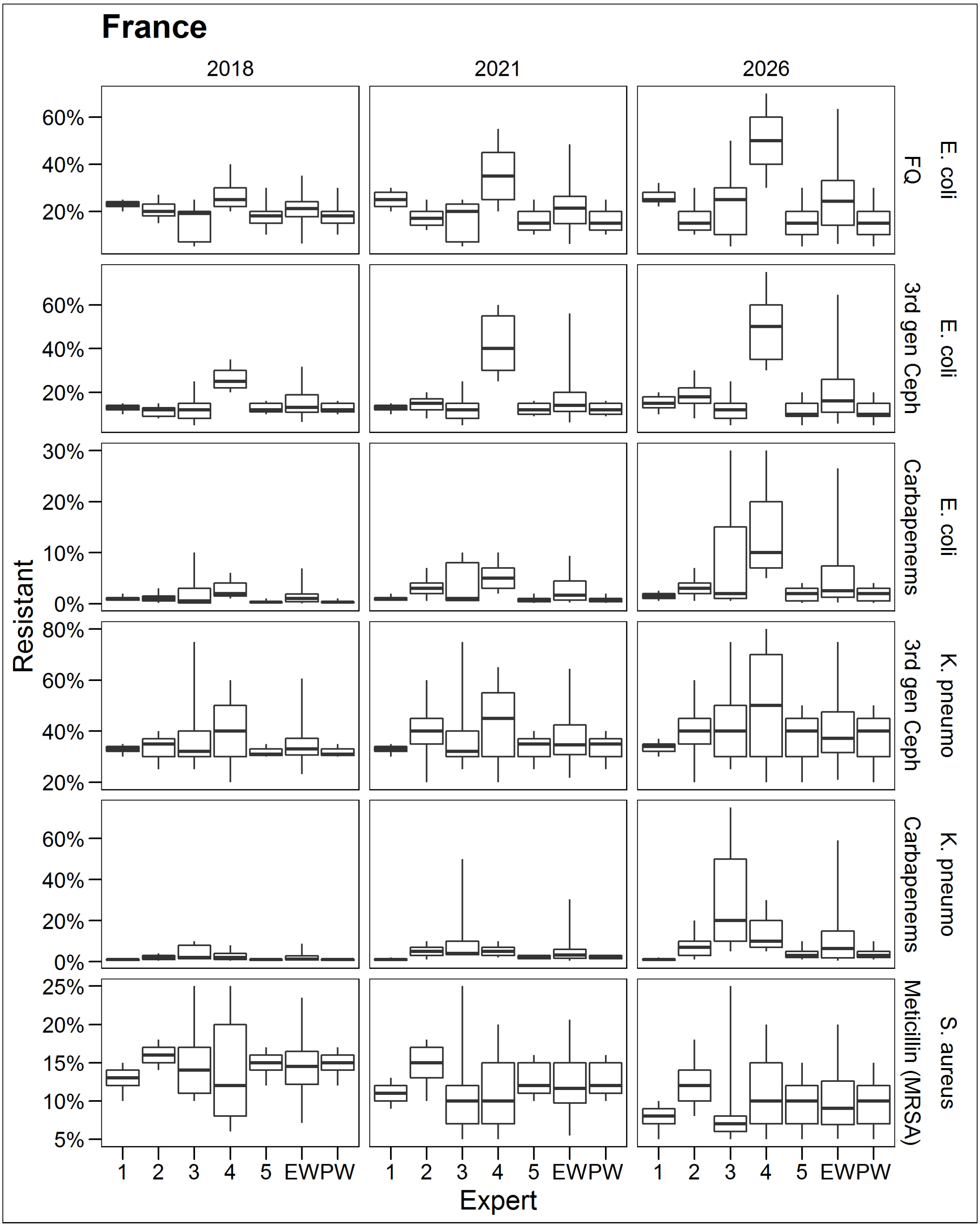

Fig F. Italy: Expert, equal-weight decision maker (EW), and performance-weight decision maker (PW) assessments for the variables of interest. Boxplots show the median estimate, 50% credible range, and 90% credible range.

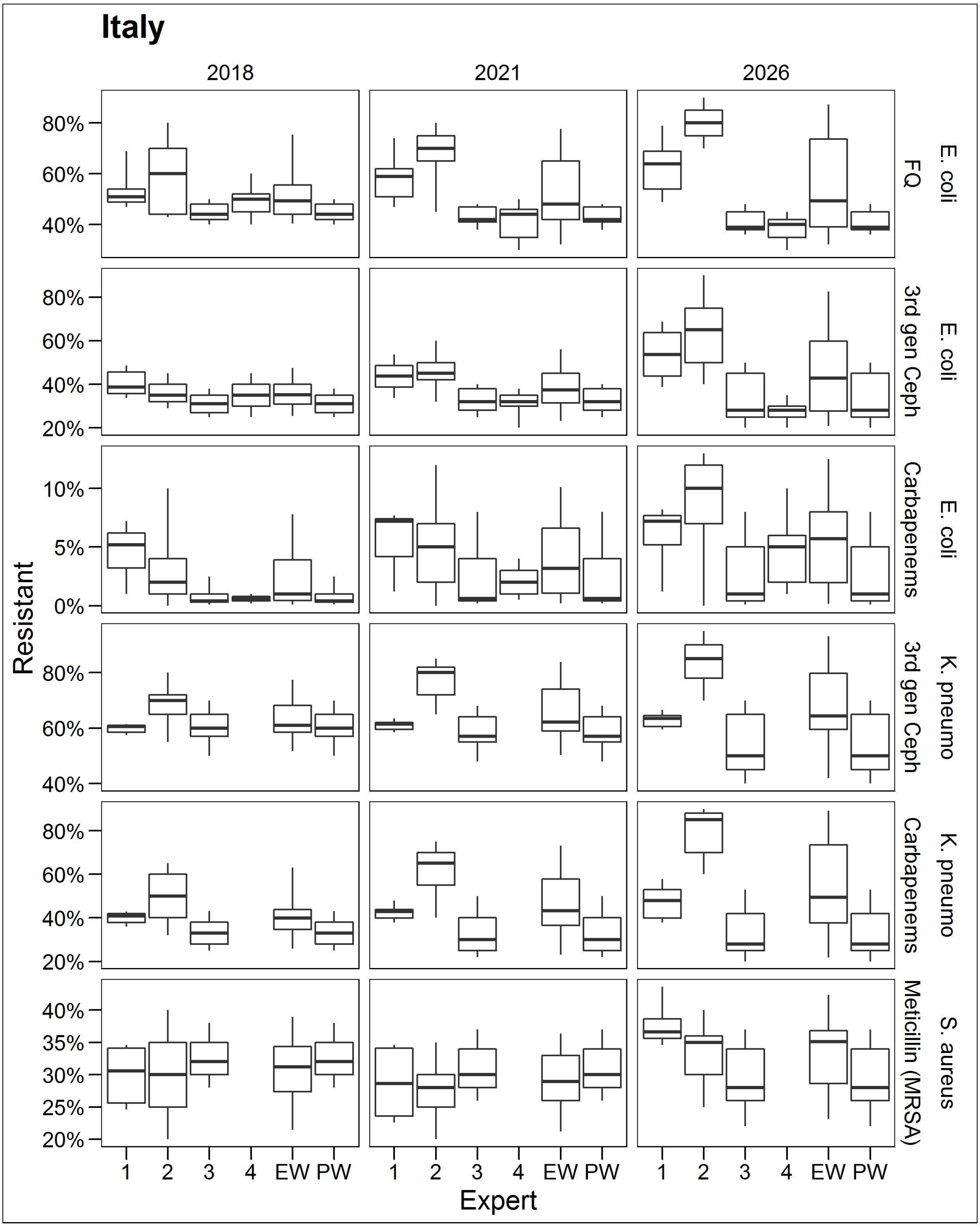

Fig G. Spain: Expert, equal-weight decision maker (EW), and performance-weight decision maker (PW) assessments for the variables of interest. Boxplots show the median estimate, 50% credible range, and 90% credible range.

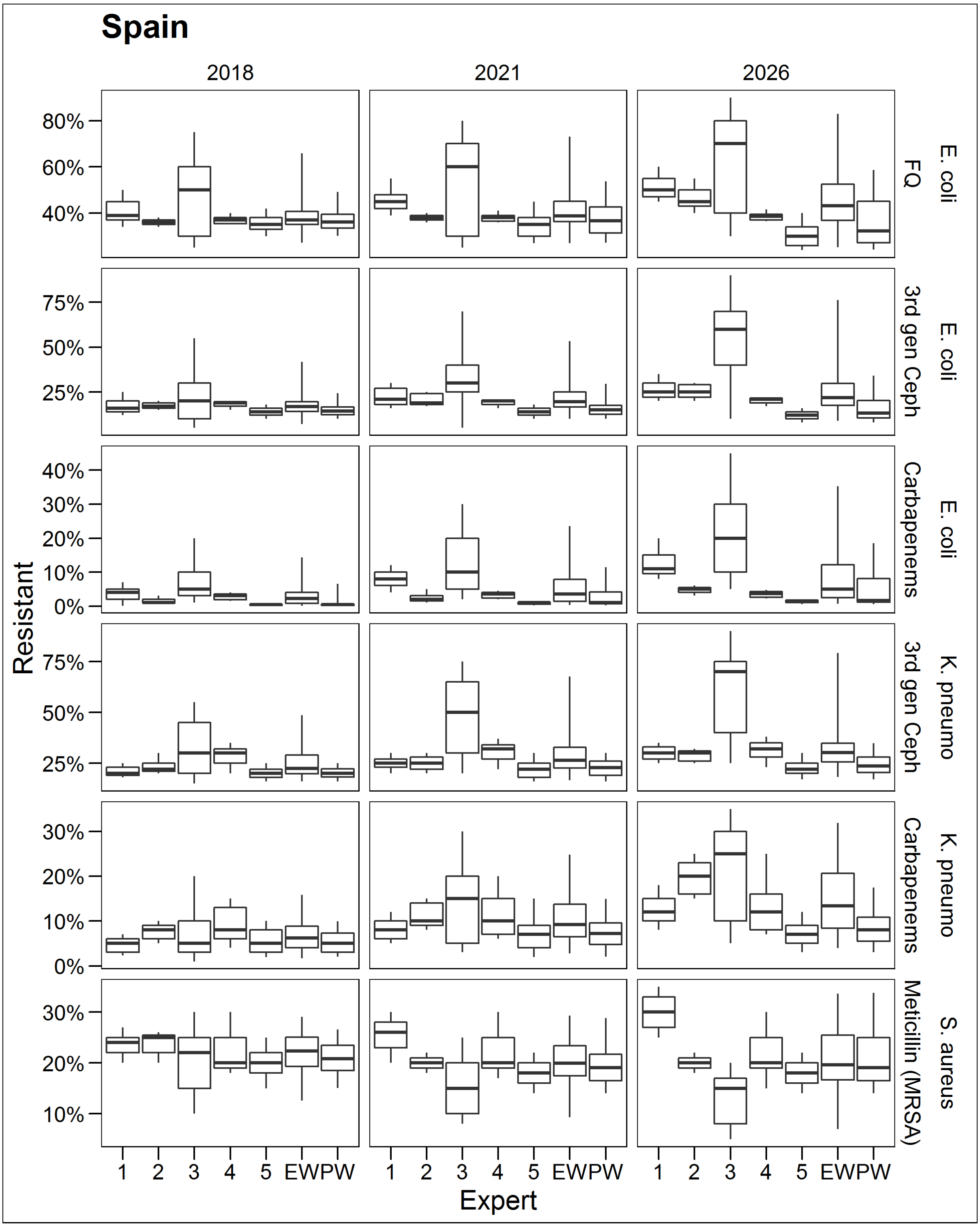

Fig H. United Kingdom: Expert, equal-weight decision maker (EW), and performance-weight decision maker (PW) assessments for the variables of interest. Boxplots show the median estimate, 50% credible range, and 90% credible range.

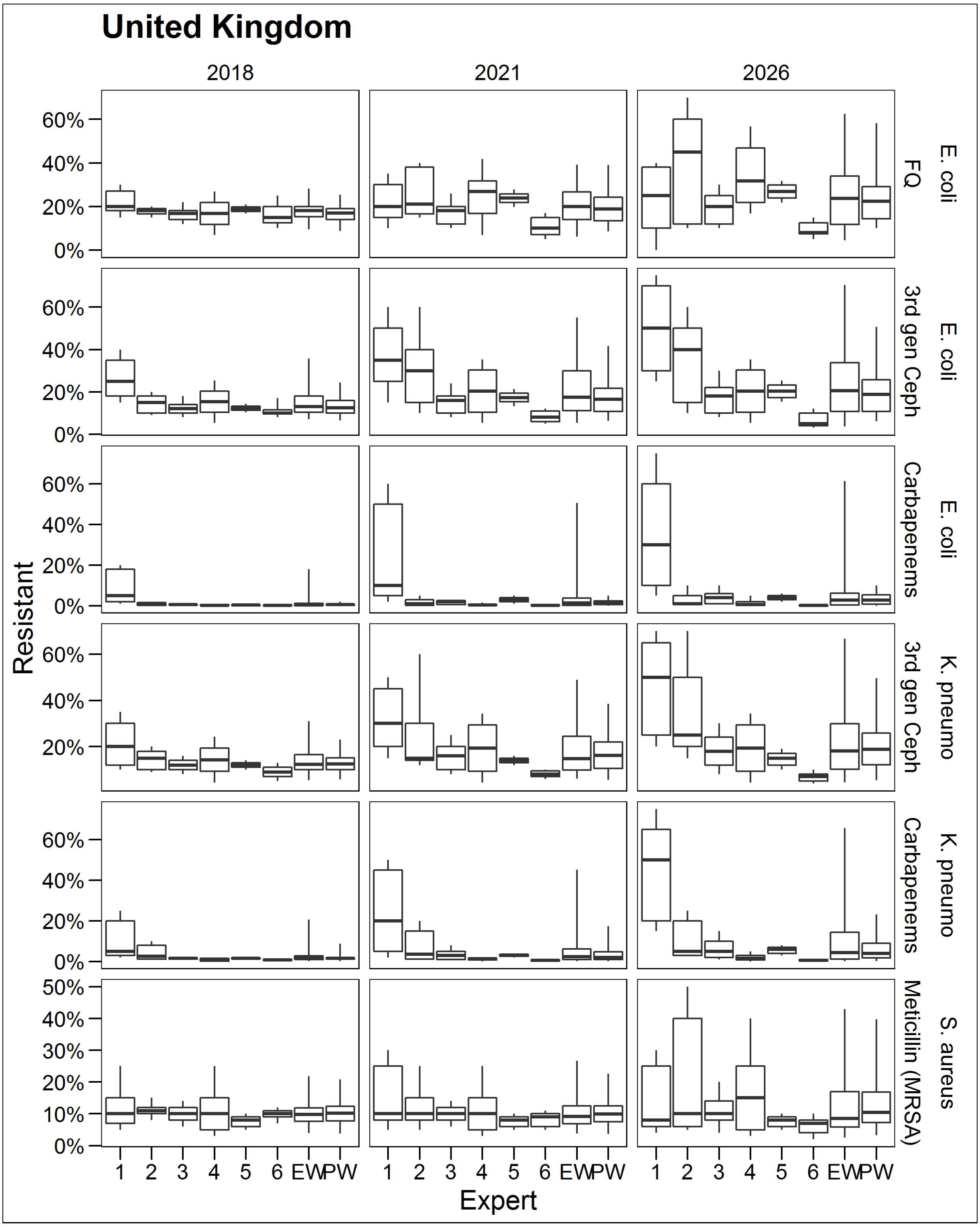

Fig I. Expert, equal-weight decision maker (EW), and performance-weight decision maker (PW) assessments for *Streptococcus pneumoniae* and intermediate susceptibility to penicillins. Boxplots show the median estimate, 50% credible range, and 90% credible range.

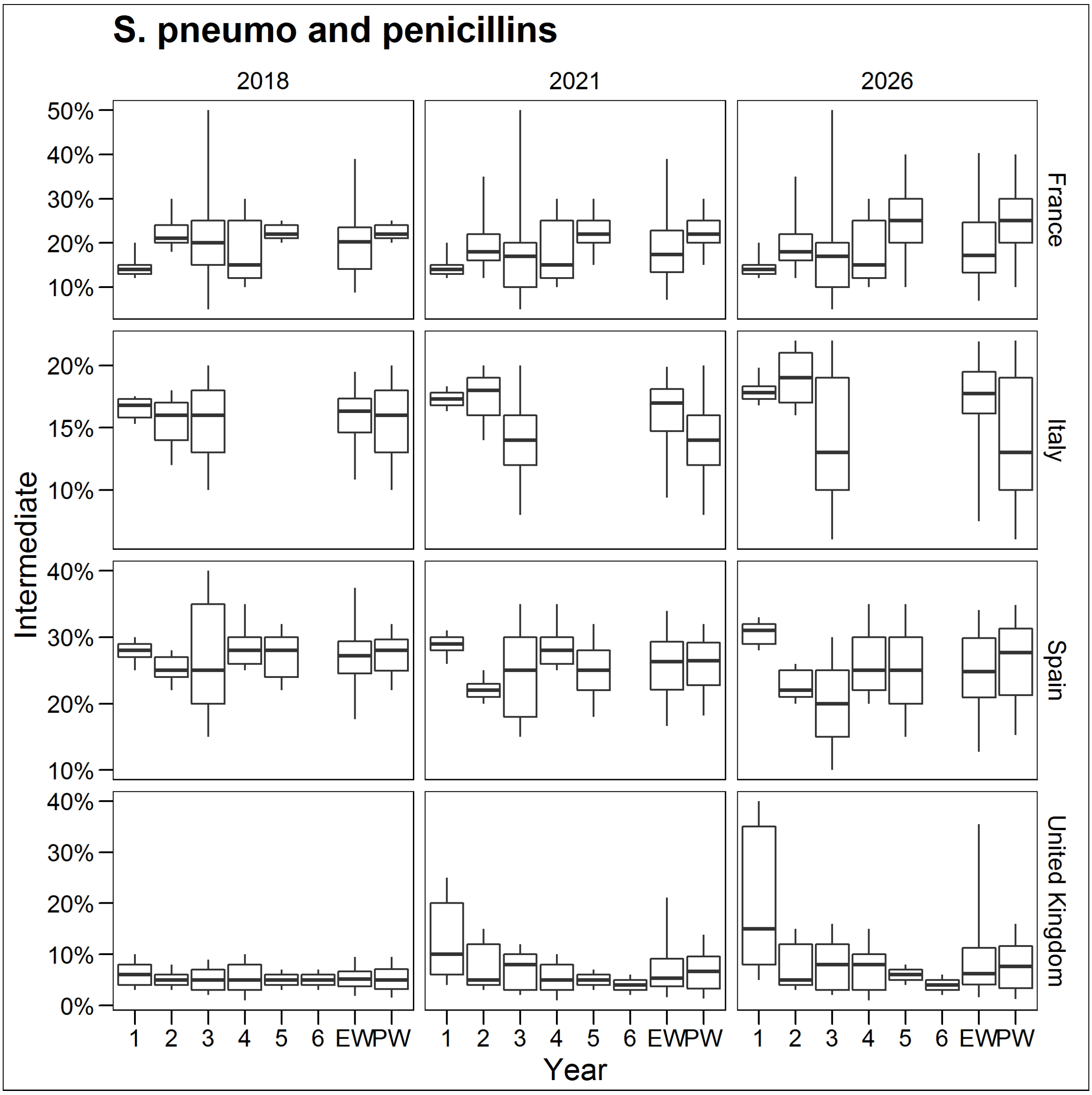

Fig J. Expert, equal-weight decision maker (EW), and performance-weight decision maker (PW) assessments for *Neisseria gonorrhoeae* resistance to third-generation cephalosporins. Boxplots show the median estimate, 50% credible range, and 90% credible range.

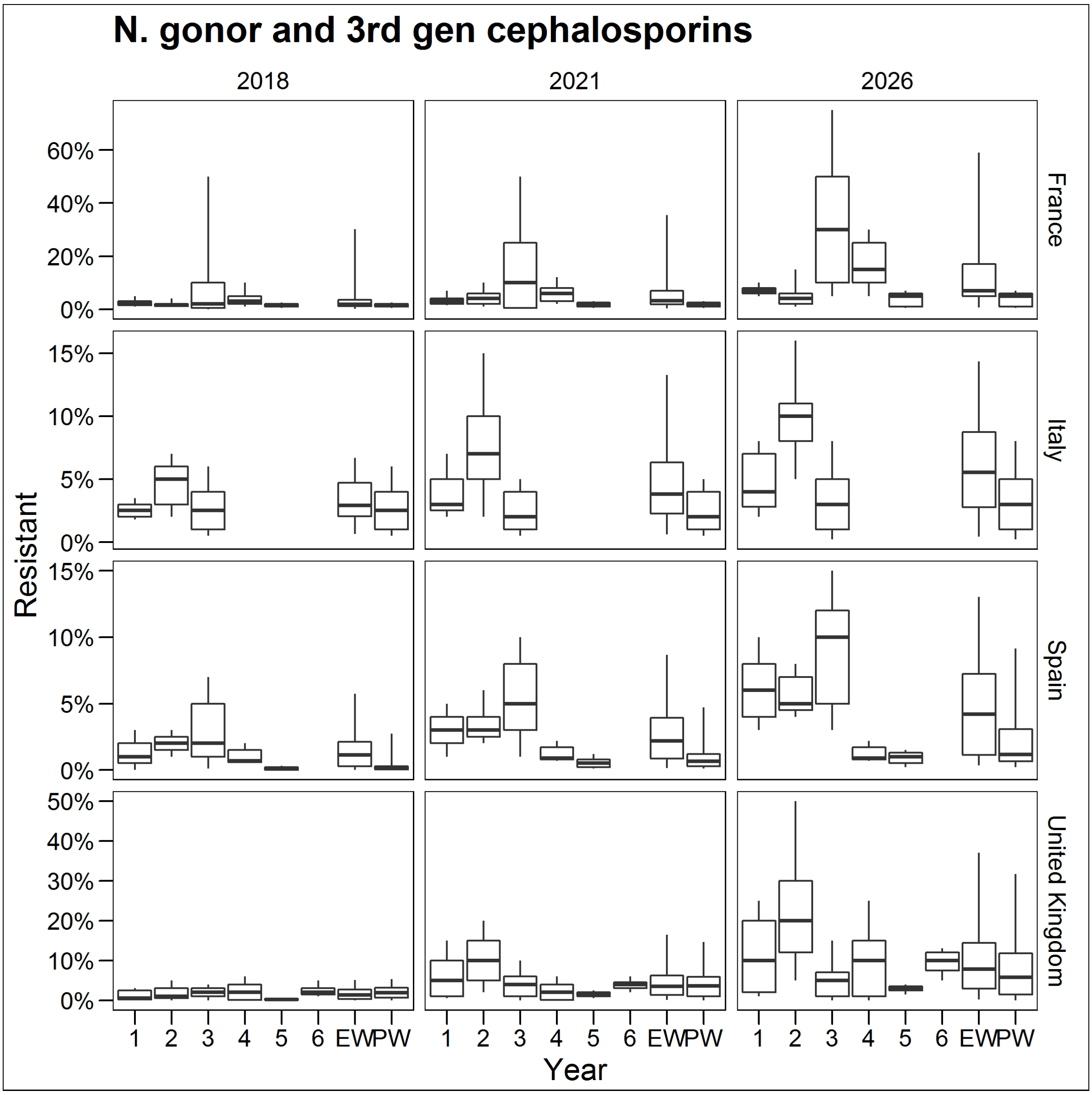

Fig K. Expert, equal-weight decision maker (EW), and performance-weight decision maker (PW) assessments for pan-drug resistant *Pseudomonas aeruginosa*. Boxplots show the median estimate, 50% credible range, and 90% credible range.

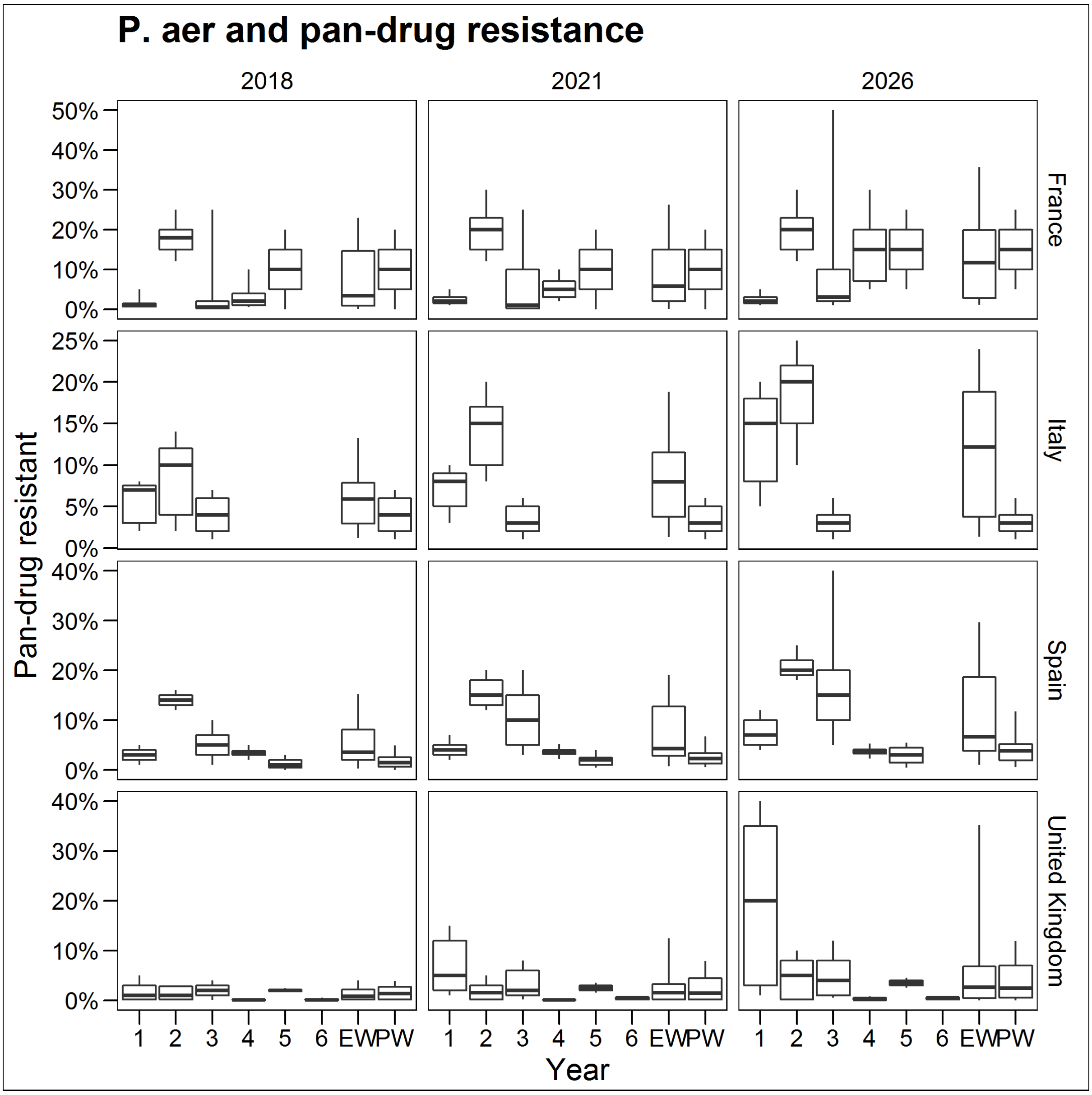

Fig L. Expert, equal-weight decision maker (EW), and performance-weight decision maker (PW) assessments for items concerning resistance rates at non-invasive sites in 2021. Boxplots show the median estimate, 50% credible range, and 90% credible range. SST = skin and soft tissue.

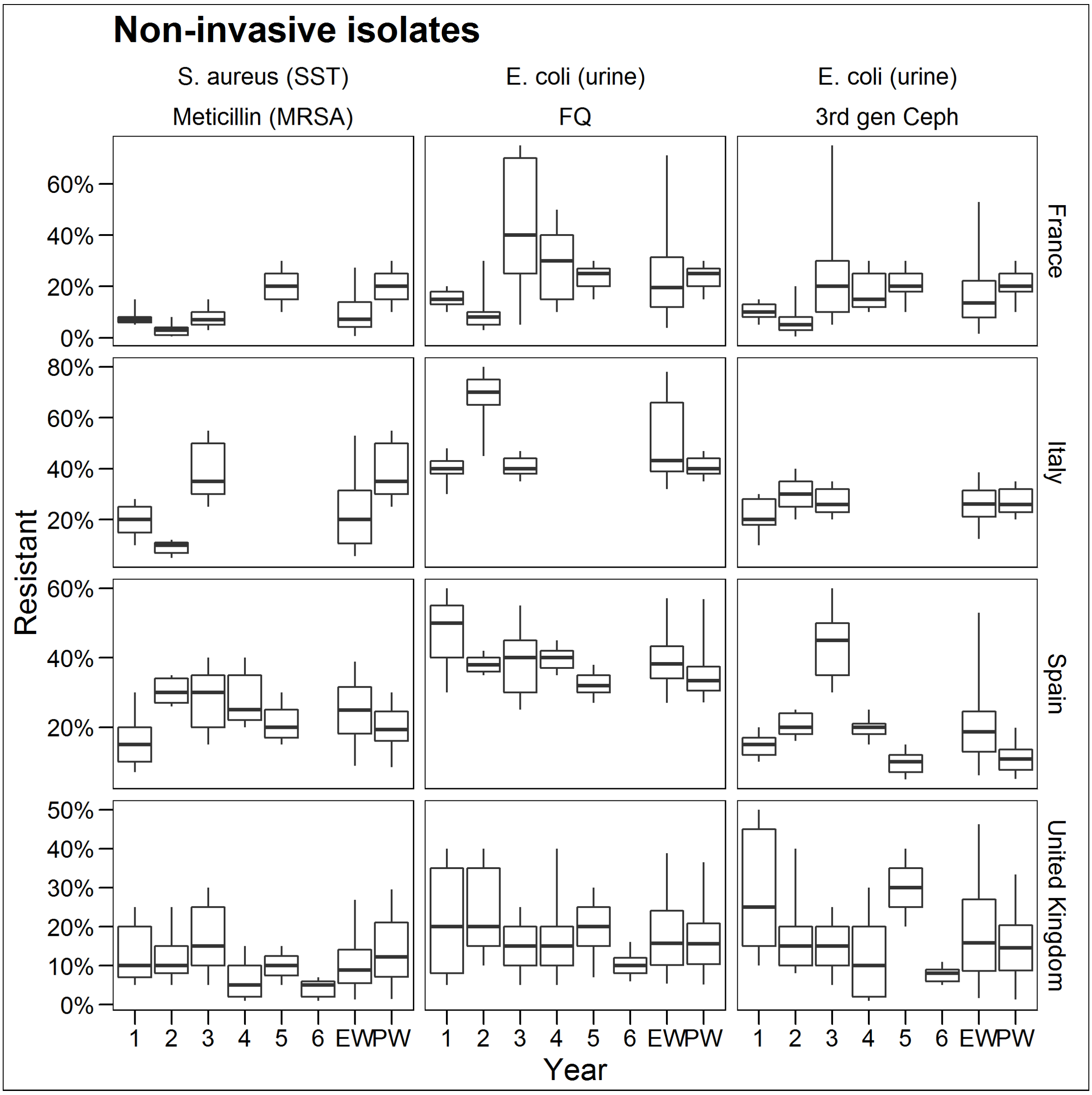

Primary statistical forecast results

Figs M-P show results from the four primary statistical forecasting models considered:

1. Exponential smoothing with additive error, no trend, and no seasonality [ETS(A,N,N)] (Fig M)
2. Exponential smoothing with additive error, additive trend, and no seasonality [ETS(A,A,N)] (Fig N)
3. Exponential smoothing with additive error, a damped additive trend, and no seasonality [ETS(A,Ad,N)] (Fig O), and
4. An autoregressive integrated moving average (ARIMA) model (Fig P).

Fig M. Results from exponential smoothing with additive error, no trend, and no seasonality [ETS(A,N,N)]. Black lines indicate the median, dark grey indicates the 50% prediction interval, and light grey indicates the 90% predication interval. Dots correspond to the median prediction for 2018, 2021, and 2026, the years assessed by the experts. Highlighted cells indicate this is the exponential smoothing model that minimizes AIC_C for that country-pathogen-antibiotic combination. This model corresponds to Panel C in Figs 2-4 of the main text.

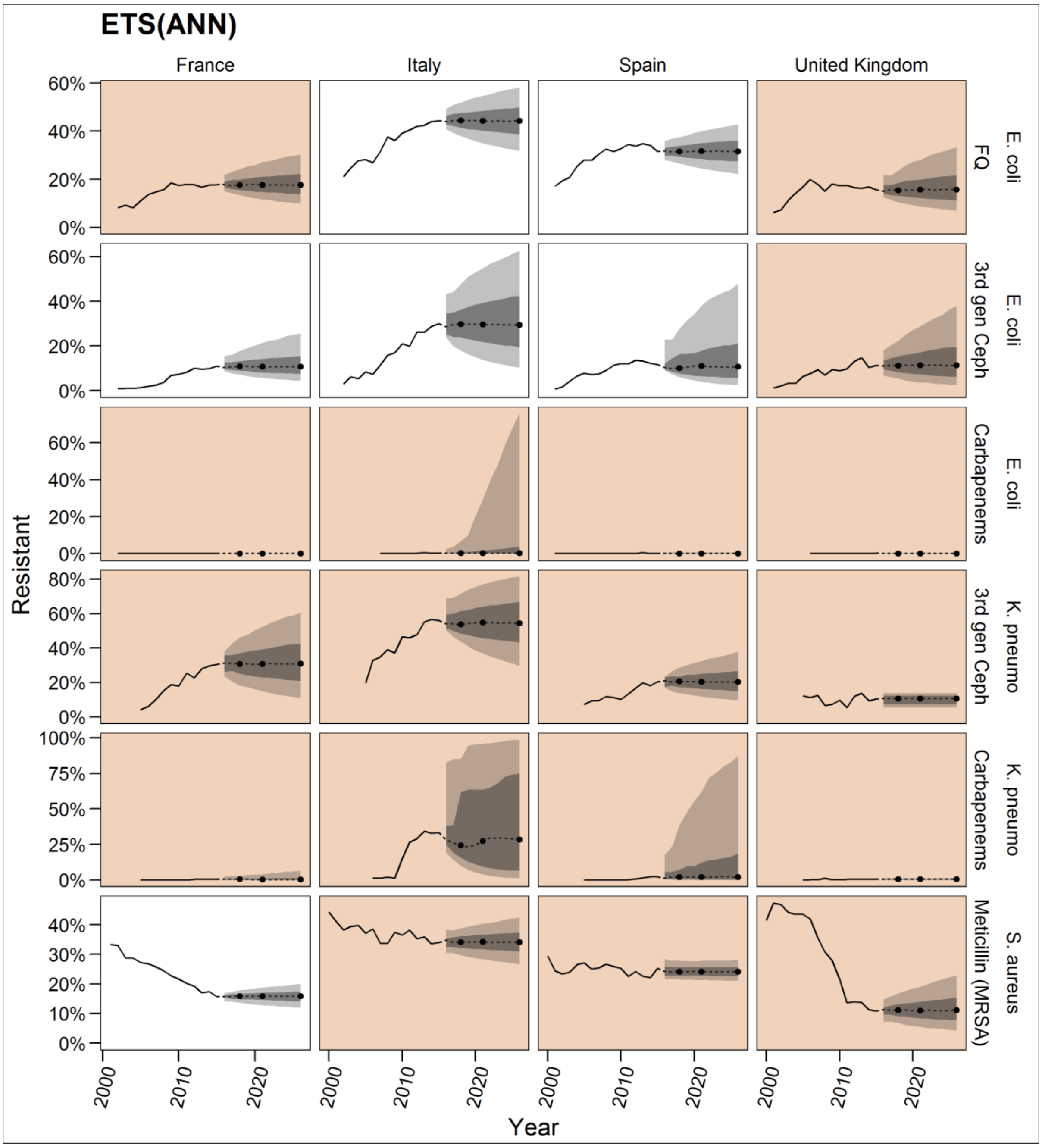

Fig N. Results from exponential smoothing with additive error, additive trend, and no seasonality [ETS(A,A,N)]. Black lines indicate the median, dark grey indicates the 50% prediction interval, and light grey indicates the 90% predication interval. Dots correspond to the median prediction for 2018, 2021, and 2026, the years assessed by the experts. Highlighted cells indicate this is the ETS model that minimizes AIC_C for that country-pathogen-antibiotic combination. This model corresponds to Panel D in Figs 2-4 of the main text.

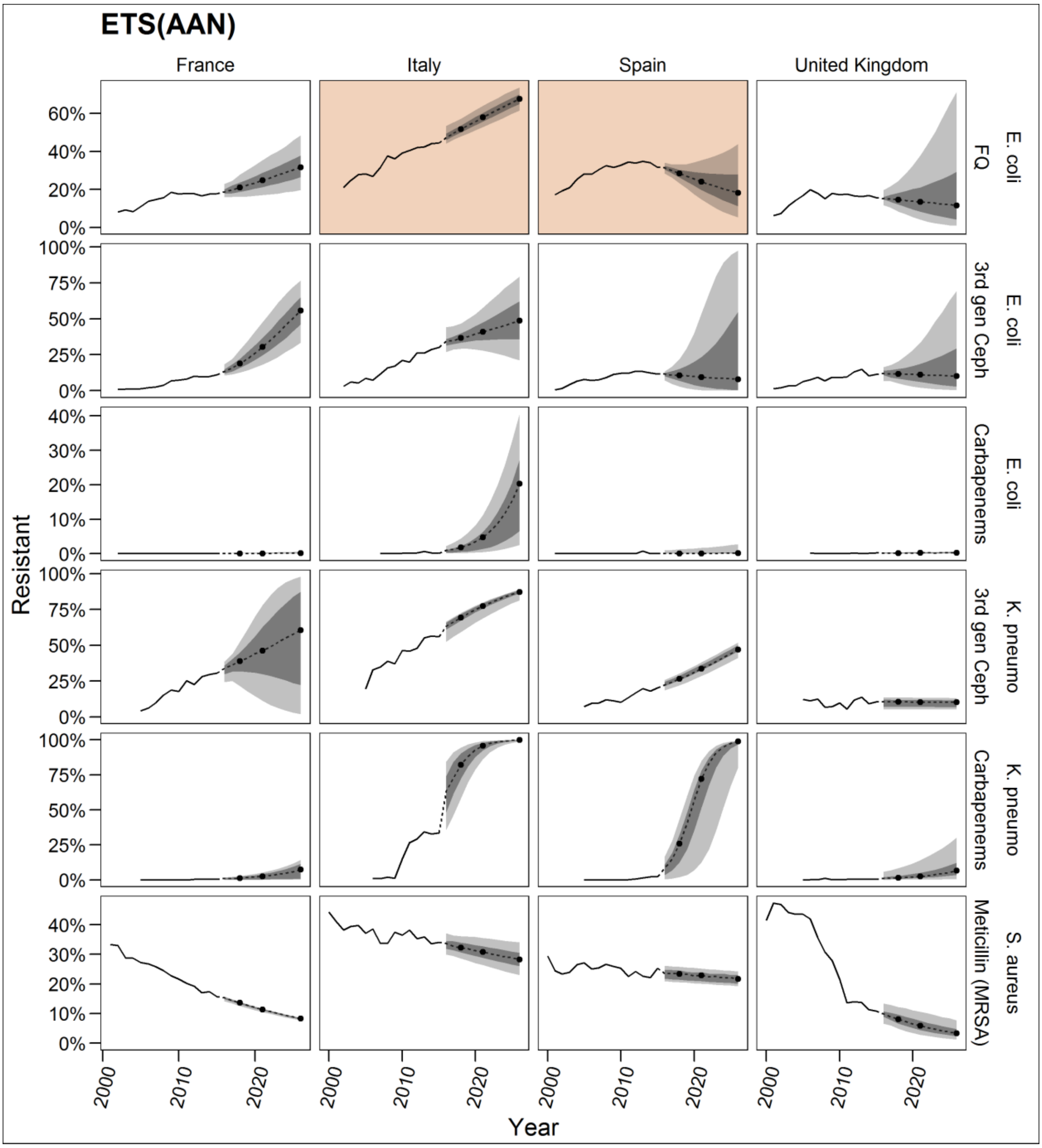

Fig O. Results from exponential smoothing with additive error, a damped additive trend, and no seasonality [ETS(A,Ad,N)]. Black lines indicate the median, dark grey indicates the 50% prediction interval, and light grey indicates the 90% predication interval. Dots correspond to the median prediction for 2018, 2021, and 2026, the years assessed by the experts. Highlighted cells indicate this is the ETS model that minimizes AIC_C for that country-pathogen-antibiotic combination. This model corresponds to Panel E in Figs 2-4 of the main text.

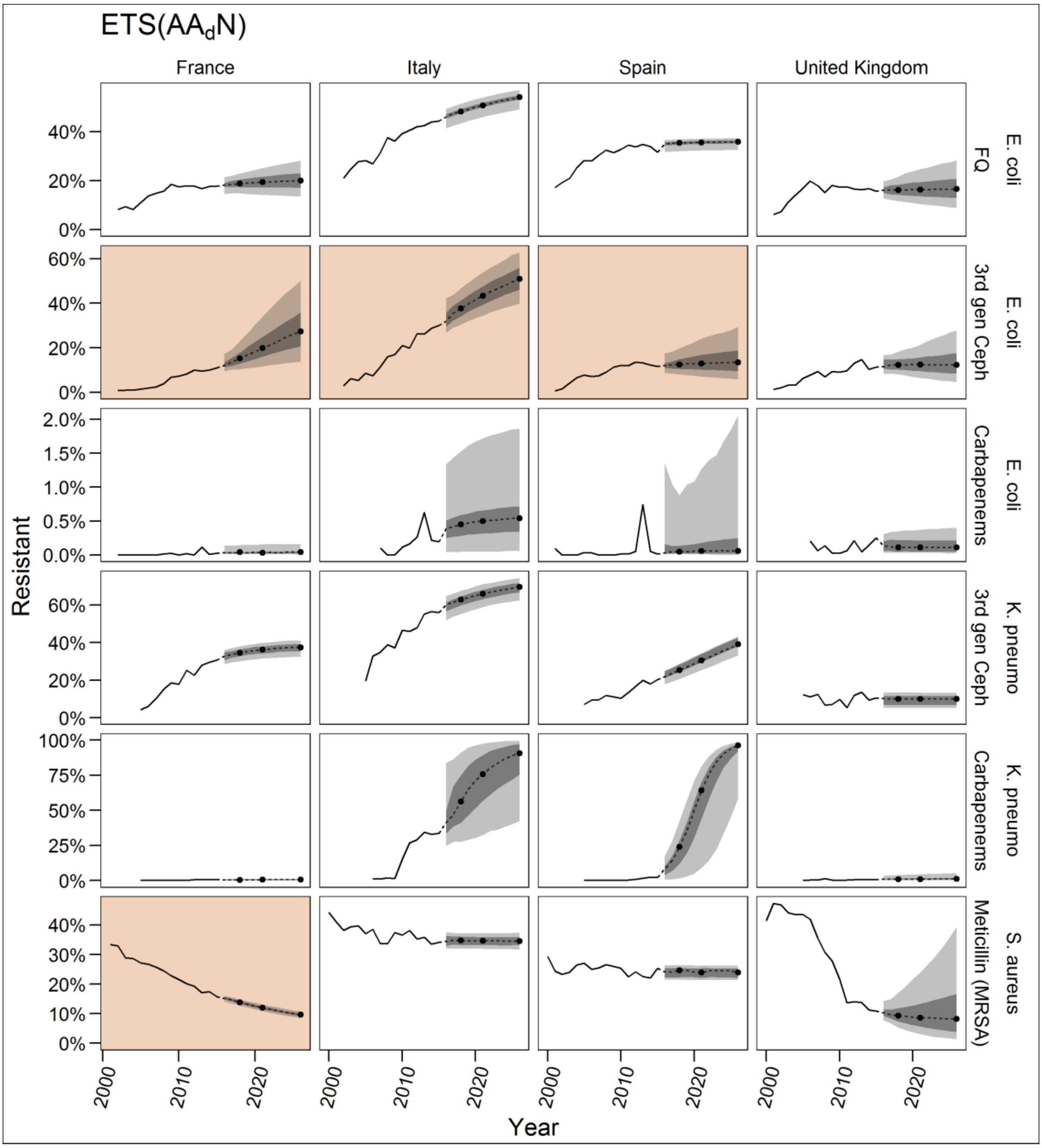

Fig P. ARIMA forecast results. Models are labelled with the ARIMA(p,d,q), values selected for that country-pathogen-antibiotic model, where p = the order of the autoregressive model, d = the degree of differencing, and q = the order of the moving average model. Black lines indicate the median, dark grey indicates the 50% prediction interval, and light grey indicates the 90% predication interval. Dots

correspond to the median prediction for 2018, 2021, and 2026, the years assessed by the experts.

This model corresponds to Panel F in Figs 2-4 of the main text.

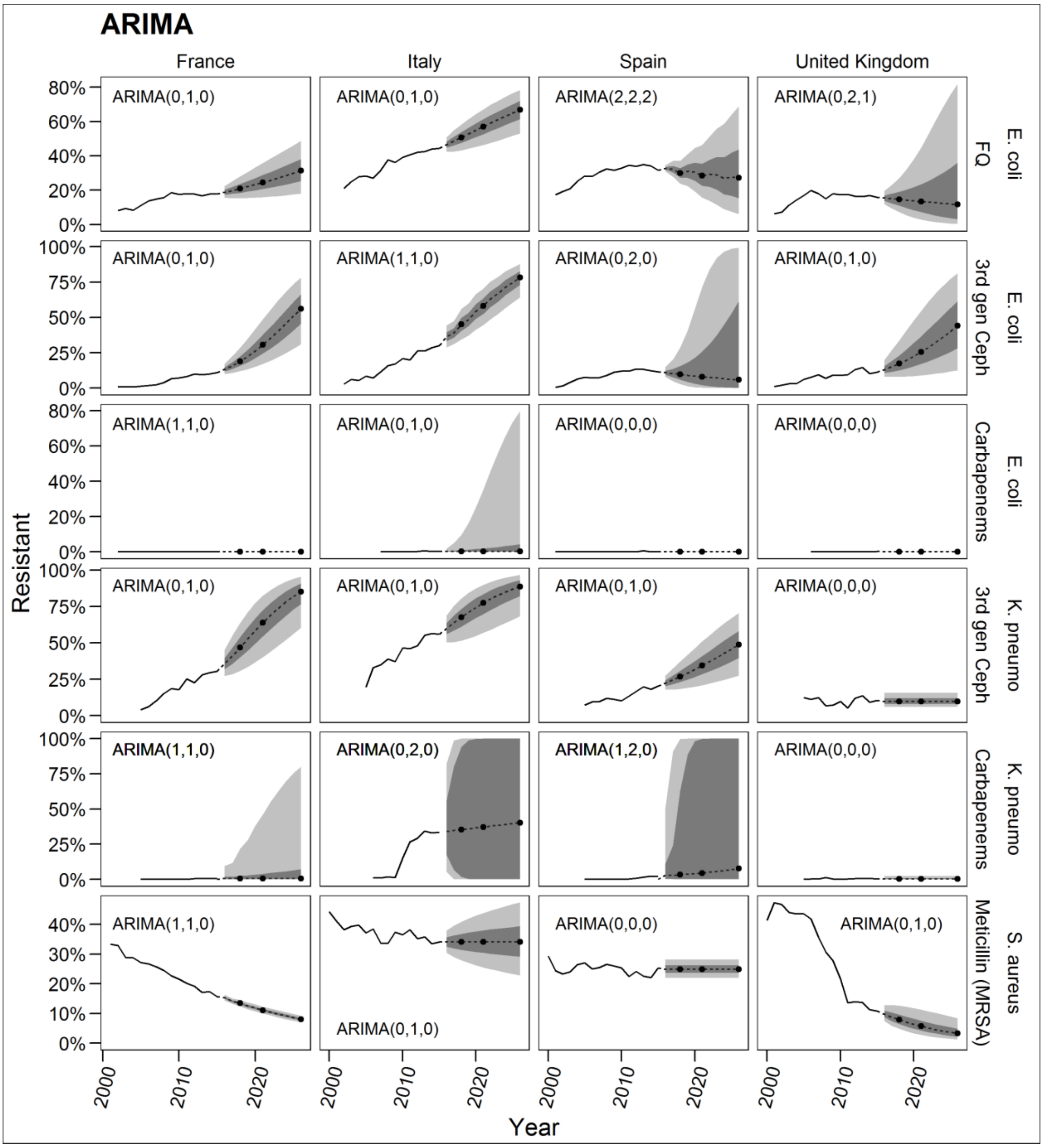

Additional statistical forecast results

In addition to the statistical forecasting models presented in the paper, we considered results from three additional forecasting models. Because combining forecasts using different methods often leads to better accuracy [5], we averaged the ARIMA and exponential smoothing models (Fig Q). We created an ARIMA model that bounds resistance such that it cannot exceed 60% (Fig R), reflecting experts' belief that resistance rates are unlikely to reach 100% as clinicians would adjust prescribing behaviour or other interventions would be undertaken before resistance hits that level. Results from this model do not differ greatly from the normal ARIMA model (Fig P), aside from the decreased maximum value. Finally, we created an exponential smoothing model without the logit transformation (Fig S). This model is equivalent to a linear extrapolation of the historical trend. The resulting projected resistance rates are less than 0% or above 100% for some combinations, demonstrating the need for a transformation or bounding the forecast. The prediction intervals from this model are typically narrower than the prediction intervals from the other statistical forecasts.

Fig Q. Hybrid forecast result: averaging ARIMA and exponential smoothing. The ETS model that minimizes AIC$_C$ was used for the exponential smoothing model. Black lines indicate the median, dark grey indicates the 50% prediction interval, and light grey indicates the 90% predication interval. Dots correspond to the median prediction for 2018, 2021, and 2026, the years assessed by the experts.

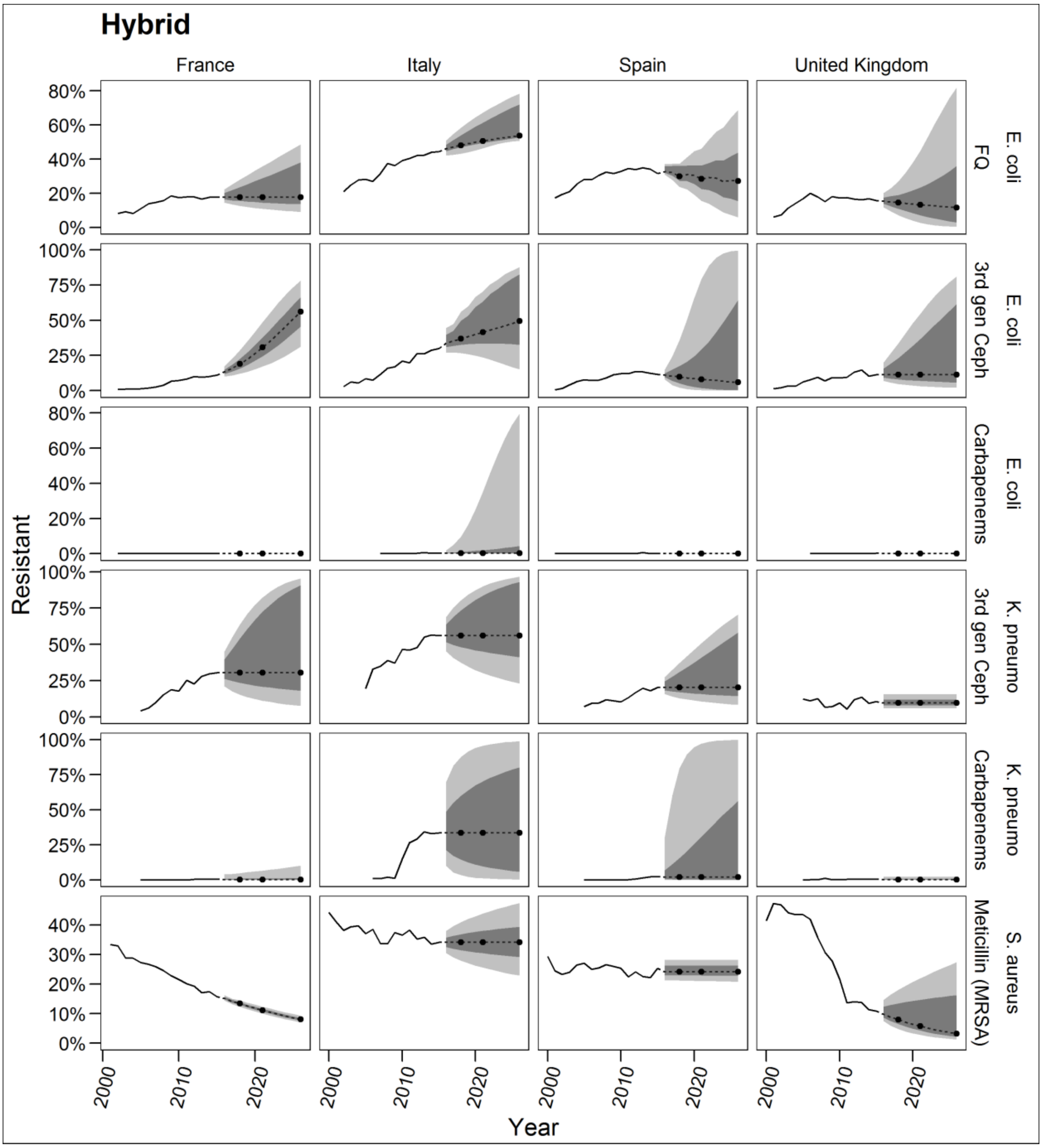

Fig R. ARIMA forecast results, bounded at 60% resistance. Black lines indicate the median, dark grey indicates the 50% prediction interval, and light grey indicates the 90% predication interval. Dots correspond to the median prediction for 2018, 2021, and 2026, the years assessed by the experts.

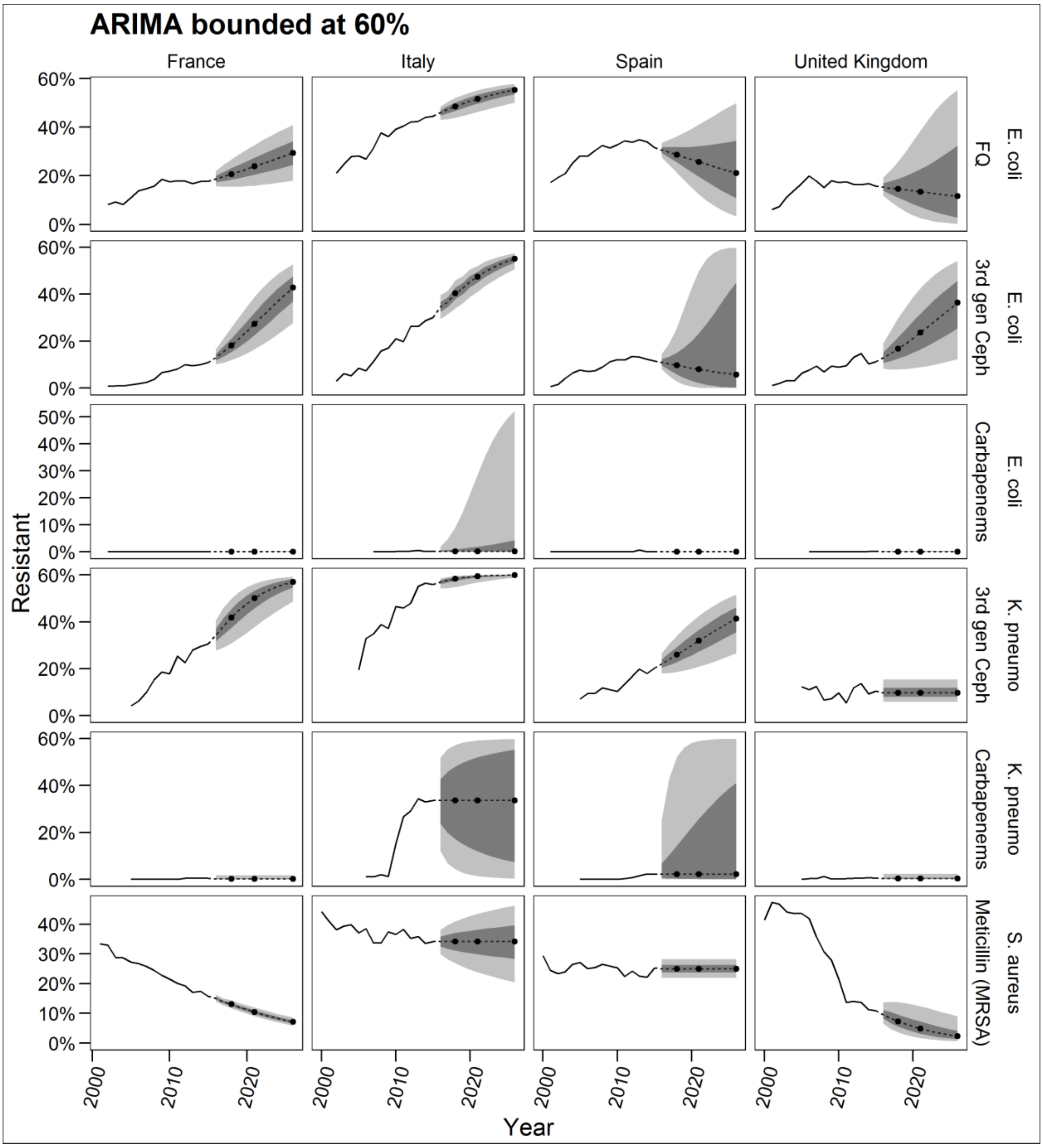

Fig S. Results from exponential smoothing with additive error, additive trend, and no seasonality [ETS(A,A,N)] and no transformation. Black lines indicate the median, dark grey indicates the 50% prediction interval, and light grey indicates the 90% predication interval. Dots correspond to the median prediction for 2018, 2021, and 2026, the years assessed by the experts.

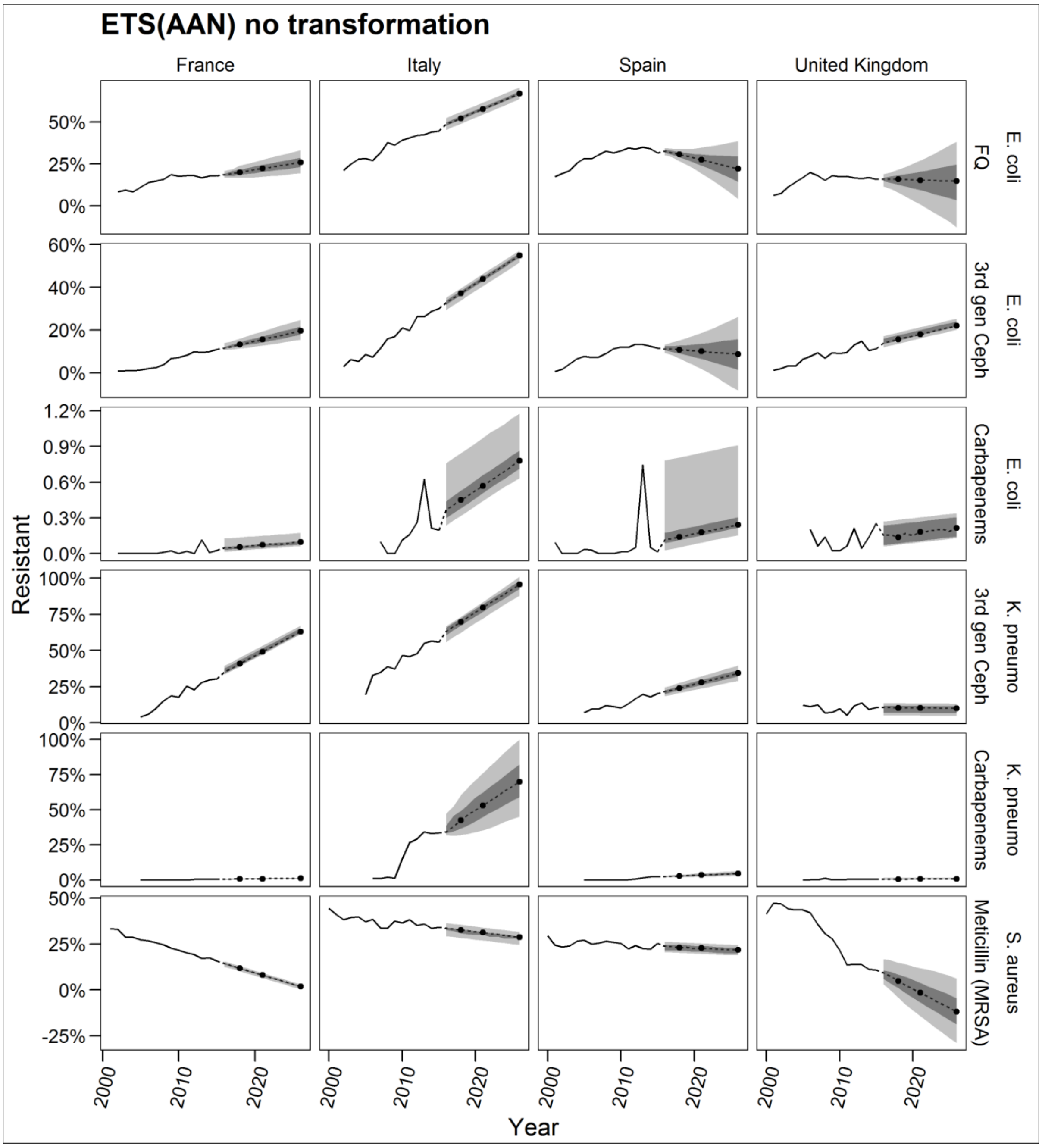

References

1. Hyndman RJ, Athanasopoulos G. Forecasting: Principles and practice [Internet]. 2014. Available: https://www.otexts.org/fpp

2. European Centre for Disease Prevention and Control. Surveillance Atlas of Infectious Diseases [Internet]. 2016 [cited 22 Jun 2017]. Available: http://atlas.ecdc.europa.eu/public/index.aspx

3. European Centre for Disease Prevention and Control. Antimicrobial resistance surveillance in Europe 2015 [Internet]. Stockholm: ECDC; 2017 Jan. Available: http://ecdc.europa.eu/en/publications/Publications/antimicrobial-resistance-europe-2015.pdf

4. European Centre for Disease Prevention and Control. Gonococcal antimicrobial susceptibility surveillance in Europe, 2014 [Internet]. Stockholm: ECDC; 2016 Aug. Available: http://ecdc.europa.eu/en/publications/Publications/gonococcal-antimicrobial-susceptibility-surveillance-Europe-2014.pdf

5. Bates JM, Granger CWJ. The Combination of Forecasts. OR. 1969;20: 451–468. doi:10.2307/3008764
